# Cytotoxic Furanoditerpenes from the Sponge *Spongia tubulifera* Collected in the Mexican Caribbean

**DOI:** 10.3390/md17070416

**Published:** 2019-07-16

**Authors:** Dawrin Pech-Puch, Jaime Rodríguez, Bastien Cautain, Carlos Alfredo Sandoval-Castro, Carlos Jiménez

**Affiliations:** 1 Centro de Investigacións Científicas Avanzadas (CICA) e Departamento de Química, Facultade de Ciencias, Universidade da Coruña, 15071 A Coruña, Spain; 2 Fundación MEDINA, Centro de Excelencia en Investigación de Medicamentos Innovadores en Andalucía, Avda. del Conocimiento 34, 18016 Granada, Spain; 3 Universidad Autónoma de Yucatán, Campus de Ciencias Biológicas y Agropecuarias, Facultad de Medicina Veterinaria y Zootecnia, Km. 15.5 Carretera Mérida-Xmatkuil, Apdo. Postal 4-116, Itzimná Mérida, Yucatán, Mexico

**Keywords:** spongian furanoditerpenes, marine sponge, *Spongia tubulifera*, ECD-TDDFT, cytotoxicity

## Abstract

Two new spongian furanoditerpenes, 3β-hydroxyspongia-13(16),14-dien-2-one (**1**) and 19-dehydroxy-spongian diterpene 17 (**2**), along with five known terpenes, the spongian furanoditerpenes 9-nor-3-hydroxyspongia-3,13(16),14-trien-2-one (**3**), 3β,19 dihydroxyspongia-13(16),14-dien-2-one (epispongiadiol) (**4**) and spongian diterpene 17 (**5**), the furanoditerpene ambliol C (**6**), and the sesterterpene scalarin (**7**), were isolated from the methanolic extract of the sponge *Spongia tubulifera*, collected in the Mexican Caribbean. The planar structures of the new compounds were elucidated by 1D/2D NMR and IR spectroscopic analysis, high resolution electrospray mass spectrometry (HRESIMS), and comparison of their spectral data with those reported in the literature. Absolute configurations were determined by comparison of the experimental electronic circular dichroism (ECD) spectrum with those calculated by time-dependent density functional theory (TDDFT). Compounds **1**, **4**, and **6** displayed weak cytotoxic activity against different human tumour cell lines.

## 1. Introduction

Specimens belonging to the genus *Spongia* have been subjected to numerous chemical investigations yielding a wide variety of C21 and other linear furanoterpenes, spongian diterpenes, scalarane sesterterpenoids, sesquiterpene quinones, sterols (including secosterols), and macrolides [1], many of which have shown biological activities including antibacterial [2,3], antiviral [4], antitumoral [5,6], and anti-inflammatory functions [7]. 

In our continuing investigations of diterpenes from marine organisms [8,9], and in particular from marine sponges [10], we have focused our attention on the sponge *Spongia tubulifera*, collected in the Mexican Caribbean, because of the cytotoxic activity found in its methanolic extract. To the best of our knowledge, the only previous reports of *S. tubulifera* were a comparative study of the fatty acids composition of specimens of this sponge collected at Ahogado Reef near La Parguera, Puerto Rico [11] and the assays of the antimicrobial activity against *Staphylococcus aureus* and *Candida albicans* of the organic extracts of specimens collected at Urabá Gulf reefs in the Colombian Caribbean [12].

In this paper, we elucidate the structures of two new spongian furanoditerpenes, 3β-hydroxyspongia-13(16),14-dien-2-one (**1**) and 19-dehydroxyspongian diterpene 17 (**2**), along with five known terpenes, **3**–**7**, and we evaluate their cytotoxic activity against a panel of five human tumour cell lines. 

## 2. Results and Discussion

**Figure 1:**
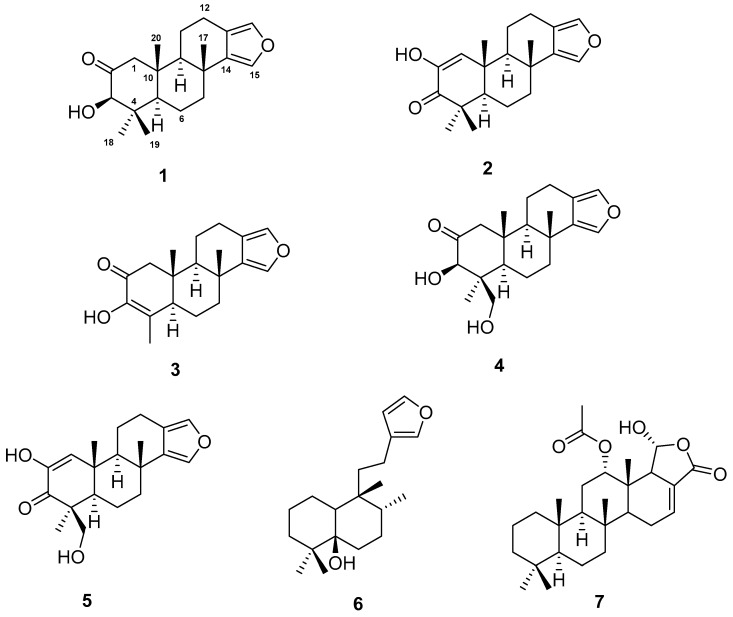
Structures of **1**–**7** isolated from *Spongia tubulifera.*

Specimens of the sponge *S. tubulifera*, collected by hand and scuba diving off the coast of the Mexican Caribbean, were extracted several times with CH_3_OH/CH_2_Cl_2_ to give an extract which showed cytotoxic activity. The organic extract was subsequently partitioned between H_2_O/CH_2_Cl_2_, and the CH_2_Cl_2_ portion was further fractionated into hexane, CH_2_Cl_2_, and aqueous methanolic fractions. The hexane fraction was submitted to silica gel flash chromatography using a gradient mixture of hexane and EtOAc to yield enriched terpene fractions that were then submitted repeatedly to reversed-phase HPLC separation (H_2_O/CH_3_OH mixtures) to yield **1**–**3** and **6**. The CH_2_Cl_2_ fraction was fractionated by solid phase extraction (SPE) with a RP-18 column using a stepped gradient from H_2_O, CH_3_OH, and CH_2_Cl_2_ to yield enriched terpene fractions that were separated by RP-HPLC using H_2_O/CH_3_OH mixtures to afford **4**, **5**, and **7** (Figure 1).

Compound **1** was obtained as a colorless white powder. The molecular formula of **1** was determined on the basis of the M^+.^ peak at *m*/*z* 316.2014, observed in its HREIM spectrum (calculated for C_20_H_28_O_3_, 316.2038, 7 degrees of unsaturation) and from its ^13^C NMR spectrum. Its IR spectrum displayed signals at 3500 and 1745 cm^−1^, suggesting the presence of a hydroxyl group and a ketone carbonyl functionality, respectively.

**Figure 2:**
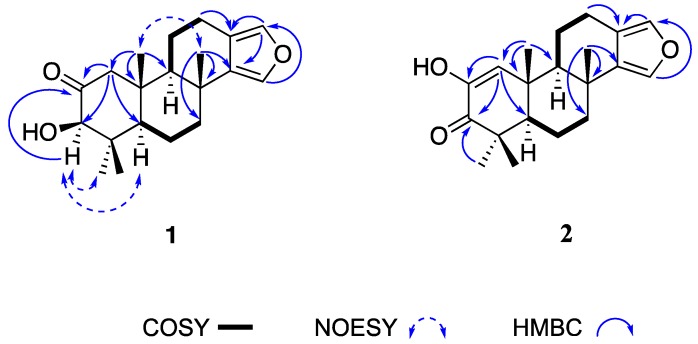
Key ^1^H-^1^H COSY, NOESY, and HMBC correlations of **1** and **2**.

The ^13^C NMR spectrum of **1** shows 20 signals (Table 1, Supplementary Material, Table S1) that, in combination with the ^1^H NMR and HSQC spectra, indicated the presence of a spongian furanditerpene bearing four tertiary methyl groups (δ_H_/δ_C_ 1.23, s/26.0; 1.21, s/29.4; 0.73, s/16.5; and 0.88, s/17.3), a 3,4-disubstituted furan ring (δ_H_/δ_C_ 7.12, s/135.3; 7.07, s/137.1; 119.4; and 136.8), one ketone carbonyl group (δ_C_ 211.1), a hydroxyl group at δ_H_ 3.48, and an oxymethine sp^3^ carbon (δ_H_/δ_C_ 3.90, d/ 83.1). Comparison of the NMR data of **1** with those of reported for other spongian furanoditerpenes, along with the HMBC correlations shown in Figure 2, indicated that **1** has a similar structure to 3α-hydroxyspongia-13(16),14-dien-2-one isolated from an unidentified *Spongia* collected in Australia [13]. The differences of the proton and carbon chemical shifts at C-3, e.g., δ_H_/δ_C_ 3.90 (d, *J* = 1.5 Hz)/ 83.1 in **1** instead of δ_H_/δ_C_ 4.36 (d, *J* = 1.5 Hz)/ 80.1 in 3α-hydroxyspongia-13(16),14-dien-2-one, suggested that they differed only in the stereochemistry at C-3, and thus, **1** must be its 3β isomer. The NOESY correlations from H-3 at δ_H_ 3.90 to H-5 at δ_H_ 1.62 and H-18 at δ_H_ 1.21 indicated that these protons were in the same face of the molecule, confirming the β-orientation of the hydroxyl group at C-3. The relative configuration of the remaining stereogenic centers in **1** was also confirmed by its NOESY correlations (Figure 2). These data indicated that **1** is a new spongian furanoditerpene derivative with a 3β-hydroxyspongia-13(16),14-dien-2-one structure. 

**Figure 3:**
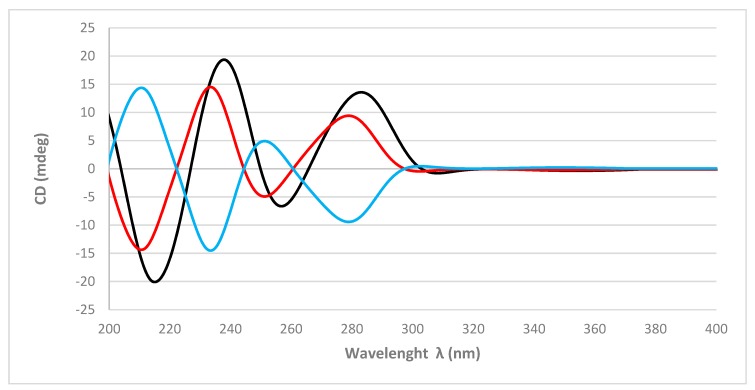
Experimental electronic circular dichroism (ECD) spectrum (black line) of **1** and calculated ECD spectrum (red line) for (3*R*,5*R*,8*R*,9*R*,10*R*)-**1** and (blue line) for (3*S*,5*S*,8*S*,9*S*,10*S*)-**1**.

The absolute configurations of the stereogenic carbons of **1** were determined by comparison of the experimental and simulated electronic circular dichroism (ECD) spectra generated by time-dependent density functional theory (TDDFT) calculations. Overall, the two possible enantiomers for **1**, (3*R*,5*R*,8*R*,9*R*,10*R*)-**1** and (3*S*,5*S*,8*S*,9*S*,10*S*)-**1**, were initially submitted to a conformational search with the Maestro Suite (Schrödinger). Four conformers were found within a 10.0 kcal/mol energy threshold from global minimum. All these conformers were geometrically optimized by a density functional theory (DFT) method at the HSEH1PBE/cc-pVDZ functions (see computational details in the experimental section). The resulting ECD spectra were combined by Boltzmann weighting to give a composite spectrum for each enantiomer. Comparison of the experimental and calculated ECD spectra for **1** showed excellent agreement with the (3*R*,5*R*,8*R*,9*R*,10*R*)-**1** enantiomer (Figure 3). Thus, the absolute configurations of C-3, C-5, C-8, C-9, and C-10 were determined as 3*R*, 5*R*, 8*R*, 9*R*, and 10*R*, respectively.

The molecular formula of **2**, isolated as a colorless white powder, was established as C_20_H_26_O_3_ based on the [M + Na]^+^ at *m*/*z* 337.1803 in its (+)-HRESIM spectrum (calculated for C_20_H_26_O_3_Na, 337.1780, 8 degrees of unsaturation) and on NMR data (Table 1, Supplementary Material, Table S2). The IR spectrum of **2** shows absorptions from the hydroxyl (3505 cm^−1^) and a conjugated ketone carbonyl (1650 cm^−1^) groups.

The 20 carbon signals observed in the ^13^C NMR spectrum of **2** along with the presence of two α-furan proton signals (δ_H_ 7.09 and 7.06) and four tertiary methyl groups (δ_H_ 1.28, 1.23, 1.22, and 1.16) in its ^1^H NMR spectrum were indicative of a spongian furanoditerpene structure. The planar structure of **2** was established by a combination of 1D and 2D NMR spectroscopy. Comparison of the NMR data of **2** with those of **1** (see Table 1) revealed that they shared the same framework at the B, C, and D rings but differed in the A-ring. Signals in the ^13^C NMR spectrum of **2** for the conjugated ketone carbonyl group at δ_C_ 201.2 (C-3) and two *sp*^2^ carbons, the non-protonated carbon at δ_C_ 144.3 (C-2) and the methine carbon at δ_C_ 128.3 (C-1), were consistent with the presence of a conjugated α,β-unsaturated ketone moiety. 

The key ^1^H-^13^C long range correlations between the olefinic proton at δ_H_ 6.54 (H-1) and the olefinic carbon at δ_C_ 144.3 (C-2), the ketone carbonyl carbon at δ_C_ 201.2 (C-3) and the carbon at δ_C_ 54.5 (C-5), along with the HMBC correlation from the methyl singlet at δ_H_ 1.22 (H-20) to the olefinic carbon at δ_C_ 128.3 (C-1) placed the α,β-unsaturated ketone in the A-ring (Figure 2). The exchangeable proton signal at δ_H_ 5.93 was indicative of an enolized α-diketone moiety in the A-ring. The NMR data for this part of the molecule (see Table 1) are in agreement with those observed for other diterpenes containing the same A-ring in the tetracyclic framework such as spongian diterpene 17 (**5**), previously reported from the nudibranch *Doriprismatica* (= *Glossodoris*) *atromarginata* [14]. The diagnostic HMBC correlations displayed by the α-furan proton signals and the methyl groups Me-17 and Me-18 displayed in Figure 2 confirm **2** as a new spongian furanoditerpene that was named 19-dehydroxy-spongian diterpene 17.

**Figure 4:**
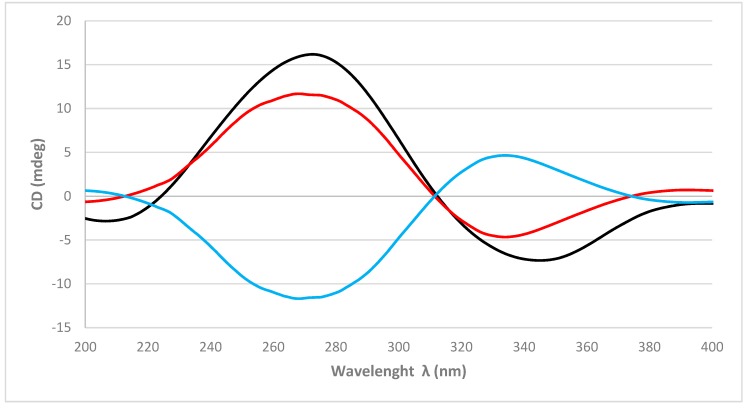
Experimental ECD spectrum (black line) of **2** and calculated ECD spectrum (red line) for (5*R*,8*R*,9*R*,10*R*)-**2** and (blue line) for (5*S*,8*S*,9*S*,10*S*)-**2**.

As in **1**, the absolute configurations of the stereogenic carbons of **2** were determined by comparison of the experimental to those generated by TDDFT on the two possible enantiomers. The two possible enantiomers for **2**, (5*R*,8*R*,9*R*,10*R*)-**2** and (5*S*,8*S*,9*S*,10*S*)-**2**, were initially submitted to a conformation search with the Maestro Suite (Schrödinger). Thus, 4 conformers were found within a 10.0 Kcal/mol energy threshold from a global minimum. All these conformers were geometrically optimized by density functional theory method at the HSEH1PBE/cc-pVDZ function (see computational details in experimental section). As shown in Figure 4, the calculated ECD spectra for the (5*R*,8*R*,9*R*,10*R*)-**2** and its experimental data were almost identical. Thus, the absolute configurations of C-5, C-8, C-9, and C-10 of **2** were determined as 5*R*, 8*R*, 9*R*, and 10*R*, respectively.

Spectral data (^1^H and ^13^C NMR, MS, ${\left[\alpha \right]}_{D}^{25}$) of **3** and **4** were identical with those reported for 19-*nor*-3-hydroxyspongia-3,13(16),14-trien-2-one (epispongiadiol) [15] and 3β-19-dihydroxyspongia-13(16),14-dien-2-one [16], respectively, isolated from an unidentified *Spongia*; while the NMR/spectroscopic data for **5** matched with those reported for spongian diterpene 17, isolated from the nudibranch *Doriprismatica* (= *Glossodoris*) *atromarginata* [14]; the NMR/spectroscopic data for **6** were identical with those reported for ambliol C, isolated from the sponge *Dysidea amblia* [17], and the NMR/spectroscopic data for **7** matched with those reported for scalarin from the sponge *Cacospongia scalaris* by Fattorusso et al. [18] and later on from *Spongia nitens* by Cimino et al. [19].

The isolated compounds were submitted to biological activity assays. MTT ((3-(4,5-Dimethylthiazol-2-yl)-2,5-diphenyltetrazolium bromide)) assays were performed on human lung carcinoma A549 ATCC® CCL-185TM, human skin melanoma A2058 ATCC® CRL-11147TM, hepatocyte carcinoma HepG2 ATCC® HB-8065TM, breast adenocarcinoma MCF7 ATCC® HTB-22TM, and pancreas carcinoma MiaPaca-2 ATCC® CRL-1420TM with doxorubicin as a positive control [20]. Compounds **1** and **4** showed a weak cytotoxic activity, while **6** exhibited the highest cytotoxic activity with IC_50_ values from 28.3 to 11.7 µM (Table 2). Previous biological studies of **4** indicated cytotoxic activity against the human tumor cell lines A549 (human lung carcinoma cells), HT-29 (human colorectal carcinoma cells), and P388 (leukemia cells lines) [21] and antiviral activity against VSV (vesicular stomalitis virus) and HSV-1 (herpes simplex virus type 1) [4]. On the other hand, it was reported that **6** induced *Artemia* sp. to death in a test of settlement and metamorphosis inhibition of larvae or juveniles [22]. Additionally, **1**–**7** did not show any significant antibacterial activity against *Acinetobacter baumannii, Pseudomonas aeruginosa, Klebsiella pneumoniae, Staphylococcus aureus*, or antiviral activity against human adenoviruses (HAdV5 and HAdV5-GFP).

## 3. Materials and Methods 

### 3.1. General Experimental Procedures

Optical rotations were measured on a JASCO DIP-1000 polarimeter, with a Na (589 nm) lamp and filter. IR spectra were measured on a FTIR Bruker Vector 22 spectrometer. ^1^H, ^13^C, and 2D NMR spectra were recorded on a Bruker Avance 500 spectrometer at 500 and 125 MHz, respectively, using CDCl_3_. Low resolution electrospray mass spectrometry (LRESIMS) and high resolution electrospray mass spectrometry (HRESIMS) experiments were performed on the Applied Biosystems QSTAR Elite system. LREIMS and HREIMS were performed on the Mass Spectrometer Thermo MAT95XP. HPLC separations were performed on the Agilent 1100 liquid chromatography system equipped with a solvent degasser, quaternary pump, and diode array detector (Agilent Technologies, Waldbronn, Germany) using a semipreparative reversed phase column Luna C18, 5 µ, 100 Å, 250 × 10 mm. Precoated silica gel plates (Merck, Kieselgel 60 F254, 0.25 mm) were used for TLC analysis, and the spots were visualized under a UV light (254 nm) or by heating the plate pretreated with H_2_SO_4_/H_2_O/AcOH (1:4:20).

### 3.2. Animal Material

The sponge *Spongia tubulifera* was collected by hand and traditional scuba diving off the coast of the Mexican Caribbean (18°48′22.17″N / 87°39′32.61″W) at depths ranging from 10 to 15 m in March 2017. Samples were frozen immediately after collection. A voucher specimen 17YUE11ST was deposited in the Phylum Porifera Gerardo Green National Collection of the Instituto de Ciencias del Mar y Limnología (ICMyL) at the National Autonomous University of México (UNAM) in Ciudad de Mexico.

### 3.3. Extraction and Isolation 

Sliced bodies of *S. tubulifera* (wet weight, 157.7 g; dry weight, 40.2 g) were exhaustively extracted with CH_3_OH-CH_2_Cl_2_ (1:1, 3 × 1.5 L) at 25 °C for 24 h each extraction. The combined extracts were concentrated under reduced pressure to give 12.0 g of a crude residue that was first partitioned between CH_2_Cl_2_/H_2_O (1:1 *v*/*v*). The resulting aqueous portion was extracted with n-butanol (200 mL) to yield the n-butanol fraction (1.44 g). The organic phase was concentrated under reduced pressure and partitioned between 10% aqueous CH_3_OH (400 mL) and hexane (2 × 400 mL) to give, after removing the solvent under reduced pressure, 526 mg of the hexane fraction. The H_2_O content (% *v*/*v*) of the methanolic fraction was adjusted to 50% aqueous CH_3_OH, and the mixture was extracted with CH_2_Cl_2_ (100 mL) to afford, after removing the solvent under reduced pressure, 1.51 g of the CH_2_Cl_2_ fraction and 2.38 g of the remaining aqueous methanolic fraction. The hexane fraction (526 mg) was subjected to a flash chromatography column on silica gel using a stepped gradient from hexane to EtOAc to give 14 fractions (FHC1-C14). Separation of the fraction FHC2, eluted with hexane/EtOAc (9:1, 93.9 mg), by RP-HPLC with a mobile phase consisting of an isocratic at 100% CH_3_OH at a flow rate of 2.0 mL/min afforded **6** (13.5 mg; *t*_R_ = 8.4 min). Separation of the fraction FHC3, eluted with hexane/EtOAc (9:1, 20.0 mg), by RP-HPLC (isocratic 100% CH_3_OH, flow rate 2.0 mL/min) gave **2** (2.6 mg; *t*_R_ = 9.7 min) and **6** (2.6 mg; *t*_R_ = 8.9 min). Separation of the fraction FHC4, eluted with hexane/EtOAc (8:2, 9.0 mg), by RP-HPLC with a mobile phase consisting of 5 min gradient from 90% to 95% of CH_3_OH in H_2_O, followed by a 10 min isocratic at 95% of CH_3_OH in H_2_O and, finally, a 5 min gradient from 95% to 100% of CH_3_OH in H_2_O at a flow rate of 2.0 mL/min yielded **2** (2.0 mg; *t*_R_ = 12.6 min). Separation of the fraction FHC5, eluted with hexane/EtOAc (8:2, 21.0 mg), by RP-HPLC with a mobile phase consisting of 5 min gradient from 90% to 95% of CH_3_OH in H_2_O, followed by a 15 min isocratic at 95% of CH_3_OH in H_2_O and, finally, a 10 min gradient from 95% to 100% of CH_3_OH in H_2_O at a flow rate of 2.0 mL/min afforded **2** (1.7 mg; *t*_R_ = 13.0 min) and **3** (1.5 mg; *t*_R_ = 12.0 min). Separation of the fraction FHC7, eluted with hexane/EtOAc (8:2, 27.2 mg), by RP-HPLC (isocratic 100% CH_3_OH, flow rate 2.0 mL/min) yielded **1** (3.0 mg; *t*_R_ = 16.8 min). Separation of the fraction FHC8, eluted with hexane/EtOAc (8:2, 20.9 mg), by RP-HPLC with a mobile phase consisting of 5 min gradient from 90% to 95% of CH_3_OH in H_2_O followed by a 15 min isocratic at 95% of CH_3_OH in H_2_O and, finally, a 1 min gradient from 95% to 100% of CH_3_OH in H_2_O at a flow rate of 2.0 mL/min afforded **1** (1.8 mg; *t*_R_ = 11.0 min). The dicloromethane fraction (1.51 g) was subjected to solid phase extraction (SPE) with RP-18 column (Merck KGaA) using a stepped gradient from H_2_O to CH_3_OH and then CH_2_Cl_2_, to give 6 fractions: H_2_O (100%), H_2_O/CH_3_OH (2:1, 1:1, and 1:2), CH_3_OH (100%), and CH_2_Cl_2_ 100%. The fraction eluted with H_2_O/CH_3_OH (1:2) was submitted to RP-HPLC separation using a mobile phase consisting of 20 min gradient from 50% to 100% of CH_3_OH in H_2_O followed by a 10 min isocratic at 100% of CH_3_OH at a flow rate of 2.0 mL/min to afford **4** (9.2 mg; *t*_R_ = 10.0 min). Separation of the fraction eluted with CH_3_OH (100%) by RP-HPLC using a mobile phase consisting of 30 min gradient from 80% to 100% of CH_3_OH in H_2_O at a flow rate of 2.0 mL/min afforded **7** (5.0 mg; *t*_R_ = 26.7 min) and **5** (3.1 mg; *t*_R_ = 29.7 min).

### 3.4. Computational Calculations

Conformational searches were performed by using the corresponding module implemented in the Maestro Quantum mechanical software. An OPLS 2005 force field with chloroform as the solvent was used, and torsional enhanced sampling with 1000 or 10,000 steps was fixed using an energy window of 10 kcal/mol. Molecular geometry optimizations were performed at the DFT theoretical level using the Gaussian 09W package firstly with a B3LYP/6-31G(d) combination and then with HSEH1PBE/cc-pVDZ auto for energy and frequency calculations. After removing redundant conformers, theoretical Boltzmann energy population-weighted ECD was calculated by using two combinations: PBEPBE/6-311++(3d,2p) or CAM-B3LYP/6-311++(3d,2p), both with 24 states. Graphical theoretical ECD curves were obtained using the open software SpecDis V.1.71 (Berlin, Germany, 2017, https:/specdis-software.jimdo.com) [23].

### 3.5. Metabolite Characterization 

**(*3R, 5R, 8R, 9R, 10R*) 3β-Hydroxyspongia-13(16),14-dien-2-one (1)**: Colorless white powder; ${\left[\alpha \right]}_{D}^{25}$ − 10.5 (c 0.1, CHCl_3_); IR (ATR neat) υ_max_ 3500, 2920, 2810, 1745, 1430, 1371, 1229, 1120, 1050, 1038, 955, 882 cm^−1^; ^1^H NMR (500 MHz) and ^13^C NMR (125 MHz) see Table 1; HREIMS *m*/*z* 316.2014 [M]^+^ (calcd. for C_20_H_28_O_3_, 316.2038).

**(*5R, 8R, 9R, 10R*) 19-Dehydroxy-spongian diterpene 17 (2)**: Colorless white powder; ${\left[\alpha \right]}_{D}^{25}$ + 18.7 (c 0.1, CHCl_3_); IR (ATR neat) υ_max_ 3505, 2950, 2855, 2325, 1650, 1430, 1370, 1230, 1120, 1050, 1038, 955, 880, cm^−1^; ^1^H NMR (500 MHz) and ^13^C NMR (125 MHz) see Table 1; (+)-HRESIMS *m*/*z* 337.1803 [M + Na]^+^ (calcd. for C_20_H_26_O_3_Na, 337.1780).

**19-nor-3-Hydroxyspongia-3,13(16),14-trien-2-one (3)**: Colorless white powder; ${\left[\alpha \right]}_{D}^{25}$ + 2.7 (c 0.1, CHCl_3_); (+)-HREIMS *m*/*z* 300.1719 [M]^+^ (calcd. for C_19_H_24_O_3_, 300.1725).

**3β, 19-Dihydroxyspongia-13(16),14-dien-2-one (epispongiadiol) (4)**: Yellow powder; ${\left[\alpha \right]}_{D}^{25}$ + 18.2 (c 0.1, CHCl_3_); (+)-HRESIMS *m*/*z* 355.1890 [M + Na]^+^ (calcd. for C_20_H_28_O_4_Na, 355.1885).

**Spongian diterpene 17 (5)**: Yellow powder; ${\left[\alpha \right]}_{D}^{25}$ − 20.4 (c 0.1, CHCl_3_); (+)-HRESIMS *m*/*z* 353.1723 [M + Na]^+^ (calcd. for C_20_H_26_O_4_Na, 353.1723).

**Ambliol C** (**6**) Yellow powder; ${\left[\alpha \right]}_{D}^{25}$ − 33.3 (c 0.1, CHCl_3_); (+)-HREIMS *m*/*z* 304.2379 [M]^+^ (calcd. for C_20_H_32_O_2_, 304.2397).

**Scalarin (7)**: Yellow powder; ${\left[\alpha \right]}_{D}^{25}$ + 41.2 (c 0.1, CHCl_3_); (+)-HRESIMS *m*/*z* 467.2767 [M + Na]^+^ (calcd. for C_27_H_40_O_5_Na, 467.2773).

### 3.6. Cytotoxic Assays

Colorimetric MTT ((3-(4,5-Dimethylthiazol-2-yl)-2,5-diphenyltetrazolium bromide)) assays were carried out to assess the cell viability of the samples against a panel of five different cancer cell lines (i.e., human lung carcinoma A549 ATCC® CCL-185TM, human skin melanoma A2058 ATCC® CRL-11147TM, hepatocyte carcinoma HepG2 ATCC® HB-8065TM, breast adenocarcinoma MCF7 ATCC® HTB-22TM, and pancreas carcinoma MiaPaca-2 ATCC® CRL-1420TM). All cells were obtained from the American Type Culture Collection (ATCC, Manassas, VA, USA). A549 cells were grown in Ham’s F12K medium with 2 mM glutamine, 10% fetal bovine serum (FBS), 100 U/mL penicillin, and 100 μg/mL streptomycin. A2058 and HepG2 were grown in ATCC formulated Eagle’s M essential medium (MEM) with 10% FBS, 2 mM l-glutamine, 1 mM sodium pyruvate, and 100 μM MEM nonessential amino acids. MCF-7 cells were grown in the previous medium supplemented with 0.01 mg/mL of bovine insulin. MiaPaca-2 cells were grown in Dulbecco’s modified Eagle’s medium (DMEM) with 10% FBS, 100U/mL penicillin, and 100 μg/mL streptomycin. The bioassays were performed as reported by Audoin et al. [20]. The cytotoxic activity was assessed after 72 h of treatment of the compound at the concentrations 0.098, 0.195, 0.391, 0.781, 1.563, 3.125, 6.250, 12.5, 25, 50, and 100 μM.

## 4. Conclusions

In summary, two new spongian furanoditerpenes, **1** and **2**, together with five known terpenes, four furanoditerpenes **3**–**6** and one sesterterpene **7**, were isolated from sponge *Spongia tubulifera* collected in the Mexican Caribbean as its metabolic components. The absolute configurations of the new compounds **1** and **2** were determined by comparison of experimental and calculated ECD spectra. Compounds **1**, **4**, and **6** displayed weak cytotoxic activity against a panel of five human tumor cell lines. This work represents the first chemical study of the secondary metabolites from *S. tubulifera*.

## Figures and Tables

**Table 1:** ^13^C (125 MHz) and ^1^H (500 MHz) NMR Data in CDCl_3_ of **1** and **2**.

Position	1		2	
	δ_C_, type	δ_H_, mult. (*J* in Hz)	δ_C_ mult.	δ_H_, mult. (*J* in Hz)
1	53.3, CH	2.67, d (12.1)	128.3, CH	6.54, s
		2.13, d (12.1)		
2	211.1, C		144.3, C	
3	83.1, CH	3.90, d (1.5)	201.2, C	
4	45.7, C		44.3, C	
5	55.0, C	1.62, m	54.5, C	1.80, m
6	18.6, CH_2_	1.66, m	19.1, CH_2_	1.67, m
		1.80, m		
7	40.7, CH_2_	1.68, m	40.4, CH_2_	1.66, m
		2.20, m		2.18, m
8	34.7, C		34.9, C	
9	56.1, CH	1.50, m	51.7, CH	1.48, dd (11.8, 1.7)
10	43.8, C		38.8, C	
11	18.9, CH_2_	1.67, m	18.8, CH_2_	1.91, dt (7.0, 1.7)
12	20.7, CH_2_	2.49, m	20.7, CH_2_	2.51, dddd (16.2, 12.2, 7.0, 1.7)
		2.82, m		2.83, ddt (16.2, 6.3, 1.5)
13	119.4, C		119.5, C	
14	136.8, C		137.3, C	
15	135.3, CH	7.12, s	135.0, CH	7.09, s
16	137.1, CH	7.07, s	137.2, CH	7.06, s
17	26.0, CH_3_	1.23, s	26.7, CH_3_	1.28, s
18	29.4, CH_3_	1.21, s	20.6, CH_3_	1.16, s
19	16.5, CH_3_	0.73, s	27.3, CH_3_	1.23, s
20	17.3, CH_3_	0.88, s	21.7, CH_3_	1.22, s
OH		3.48, d (1.5)		5.93, s

**Table 2:** Cytotoxic Activity Data (IC_50_ in µM) of **1**, **4**, and **6** ^a^.

Tumour Cell Lines	Compound			
	1	4	6	Doxorubicin
A549 (lung)	88.1 ± 7.9	73.7 ± 6.3	28.3 ± 2.1	0.4 ± 0.2
A2058 (skin)	71.4 ± 2.5	53.9 ± 0.6	22.9 ± 0.7	0.1 ± 0.1
HepG2 (hepatocyte)	91.3 ± 15.8	60.1 ± 5.0	24.3 ± 0.2	0.1 ± 0.1
MCF-7 (breast)	nd	nd	19.9 ± 3.3	5.1 ± 0.5
MiaPaca-2 (pancreas)	90.0 ± 44.8	nd	11.7 ± 0.9	6.6 ± 0.5

^a^ IC_50_, compound concentration that produces 50% inhibition on cell growth as compared to control cells. nd: not detected.

## Supplementary Materials

The following are available online at https://www.mdpi.com/1660-3397/17/7/416/s1, Figures S1–S6: NMR spectra of **1** in CDCl_3_, Figure S7: HREIMS of **1**, Figures S7–S13: NMR spectra of **2** in CDCl_3_, Figure S14: (+)-HRESIMS of **2**, Figures S15–S23: NMR spectra of **3**–**7** in CDCl_3_, Table S1: NMR data of **1** in CDCl_3_, Table S2: NMR data of **2** in CDCl_3_.
