# Supplementary material for: Cytotoxic Furanoditerpenes from the Sponge Spongia tubulifera Collected in the Mexican Caribbean
Source: Mar Drugs. 2019 Jul 16;17(7):416. doi: 10.3390/md17070416 (PMC6669439; doi:10.3390/md17070416)
Supplement: Supplementary file 1 [file marinedrugs-17-00416-s001.pdf]

Supplementary Materials

Cytotoxic Furanoditerpenes from the Sponge *Spongia tubulifera* Collected in the Mexican Caribbean

Dawrin Pech-Puch[1], Jaime Rodríguez[1*], Bastien Cautain[2], Carlos A. Sandoval-Castro[3] and Carlos Jiménez[1*]

[1] Centro de Investigacións Científicas Avanzadas (CICA) e Departamento de Química, Facultade de Ciencias, Universidade da Coruña, 15071 A Coruña, Spain
[2] Fundación MEDINA, Centro de Excelencia en Investigación de Medicamentos Innovadores en Andalucía, Avda. del Conocimiento 34, 18016 Granada, Spain
[3] Universidad Autónoma de Yucatán, Campus de Ciencias Biológicas y Agropecuarias, Facultad de Medicina Veterinaria y Zootecnia, Km. 15.5 Carretera Mérida-Xmatkuil, Apdo. Postal 4-116, Itzimná Mérida, Yucatán, México

INDEX

Table S1. NMR data of **1** in $CDCl_3$....……………………………..…….……..…………S3
Figure S1-S6. NMR spectra of **1** in $CDCl_3$…………..……..………………..…….......S4-S6
Figure S7. HREIMS of **1**…….…………...……………..……..……..……………..…...S7
Table S2. NMR data of **2** in $CDCl_3$…………...……………...………………...….…………S8
Figure S8-S13. NMR spectra of **2** in $CDCl_3$………………………..…. …….……S9-S11
Figure S14. (+)-HRESIMS of **2**……………...….....................………………………...S12
Figure S15-S16. NMR spectra of **3** in $CDCl_3$…………………..……..… …….……….S13
Figure S17-S18. NMR spectra of **4** in $CDCl_3$…………..…….……….…..… …….……S14
Figure S19-S20. NMR spectra of **5** in CH_3OH ……………………….…..… ……......…S15
Figure S20-S21. NMR spectra of **6** in $CDCl_3$………………………..……….….……S16
Figure S22-S23. NMR spectra of **7** in $CDCl_3$………………………..….…...….………S17

Table S1. NMR data of **1** in $CDCl_3$ (125 MHz for ^{13}C and 500 MHz for ^{1}H).

no.	δ_C type	δ_H, mult. (*J* in Hz)	COSY	HMBC	NOESY
1	53.3, CH	2.67, d (12.1)-<br>2.13, d (12.1)		2, 3, 5, 10, 20	3, 9, 11, 20
2	211.1, C				
3	83.1, CH	3.90, d (1.5)	OH	2, 4, 18, 19	1, 5, 18
4	45.7, C				
5	55.0, C	1.62, m	6	1, 18, 19	3
6	18.6, CH_2	1.66, m-1.80, m	5, 7		18
7	40.7, CH_2	1.68, m-2.20, m	6		17
8	34.7, C				
9	56.1, CH	1.50, m	11		1
10	43.8, C				
11	18.9, CH_2	1.67, m	9, 12		1
12	20.7, CH_2	2.49, m-2.82, m	11	13	
13	119.4, C				
14	136.8, C				
15	135.3, CH	7.12, s		13, 16	
16	137.1, CH	7.07, s		13, 14	
17	26.0, CH_3	1.23, s		7, 8, 14	7, 20
18	29.4, CH_3	1.21, s		3, 4, 5, 19	6, 19
19	16.5, CH_3	0.73, s		3, 4, 5, 18	18
20	17.3, CH_3	0.88, s		1, 9, 10	1, 17
OH		3.48, d (1.5)	3		

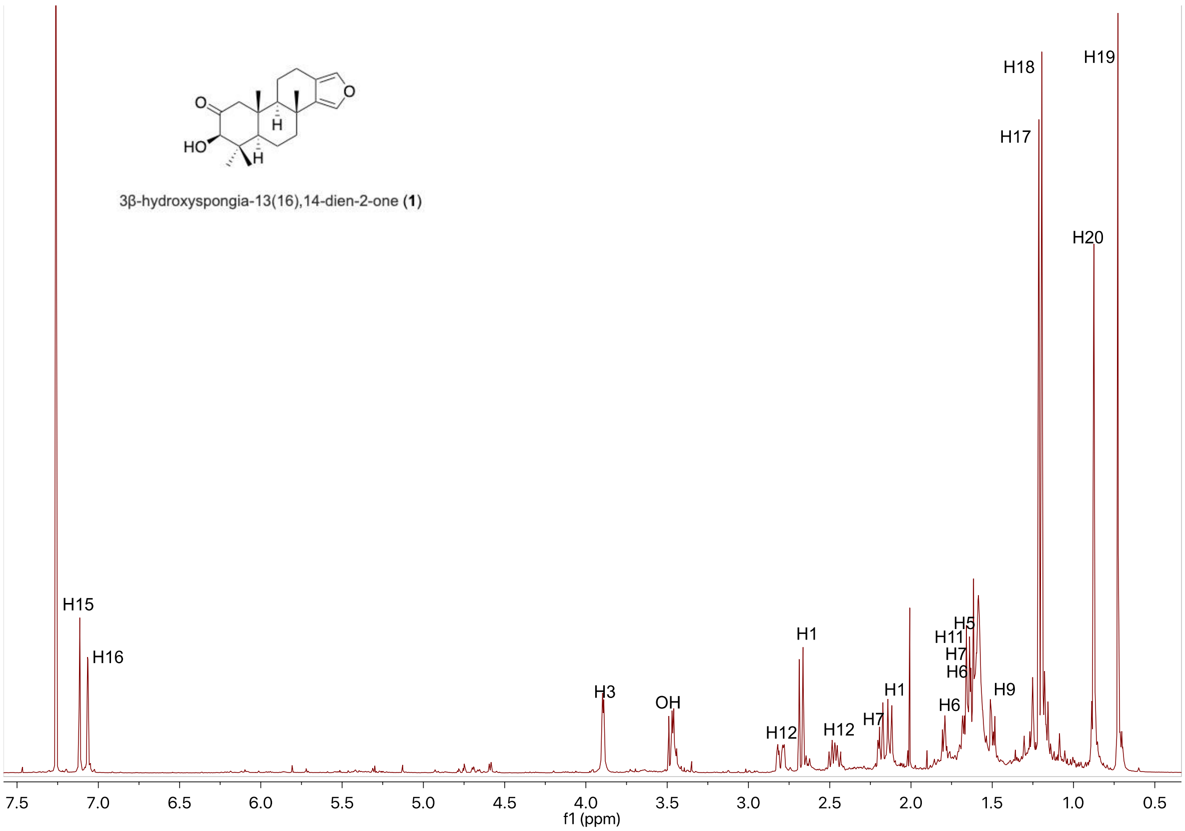

Figure S1. ^{1}H NMR spectrum of **1** (500 MHz, $CDCl_3$).

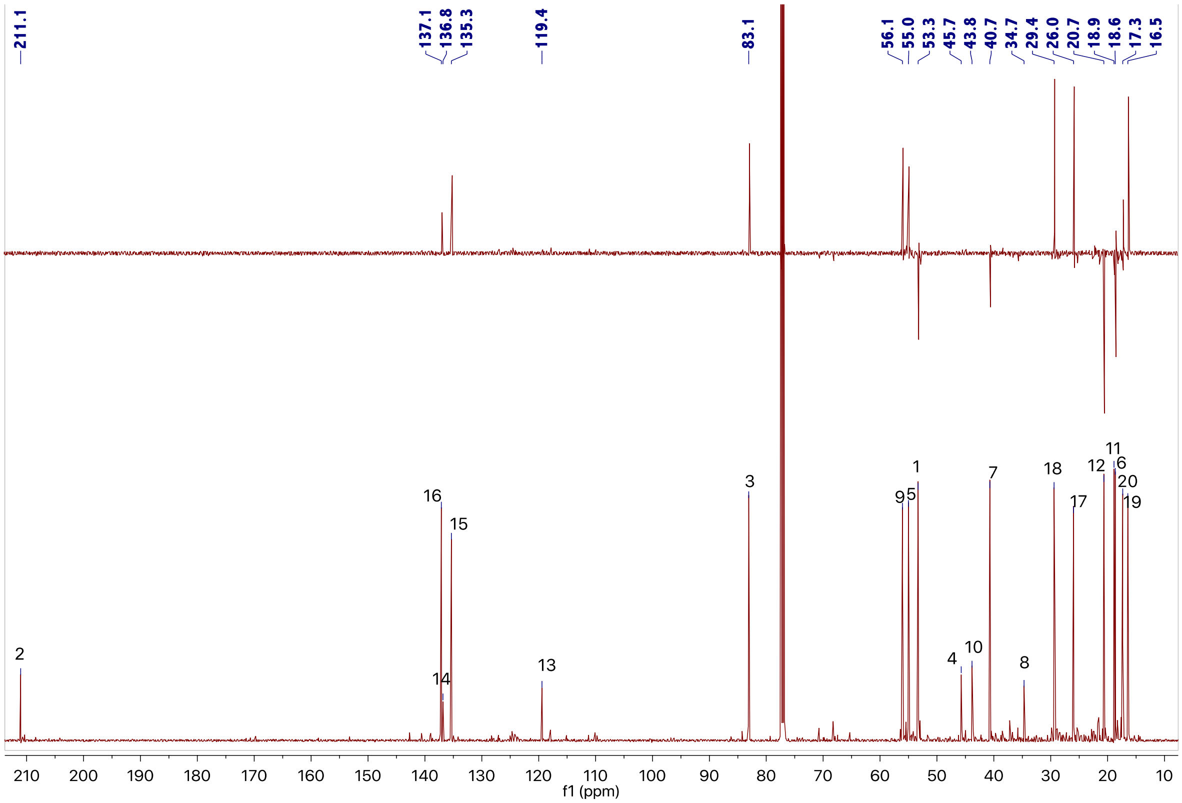

Figure S2. ^{13}C NMR and DEPT-135 spectrum of **1** (125 MHz, $CDCl_3$).

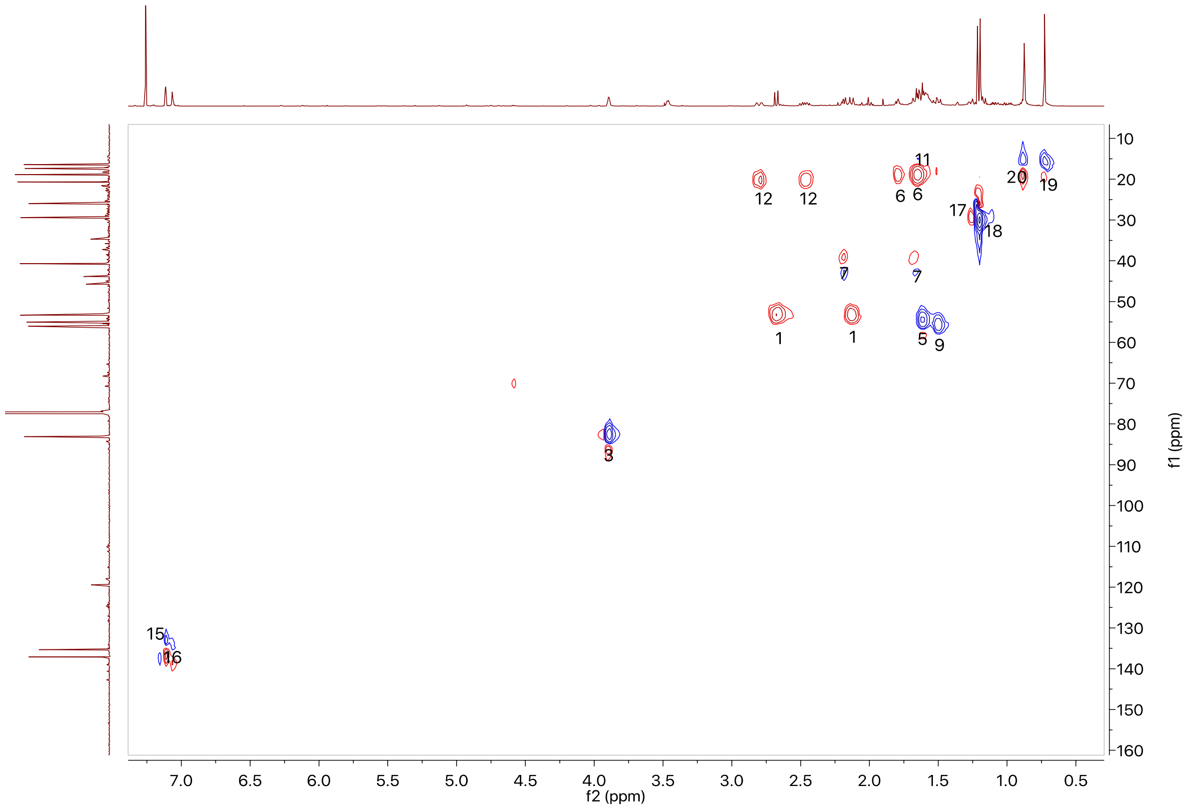

Figure S3. HSQC spectrum of **1** (500 MHz, $CDCl_3$). CH_2: blue cross-peaks and CH or CH_3: red cross-peaks.

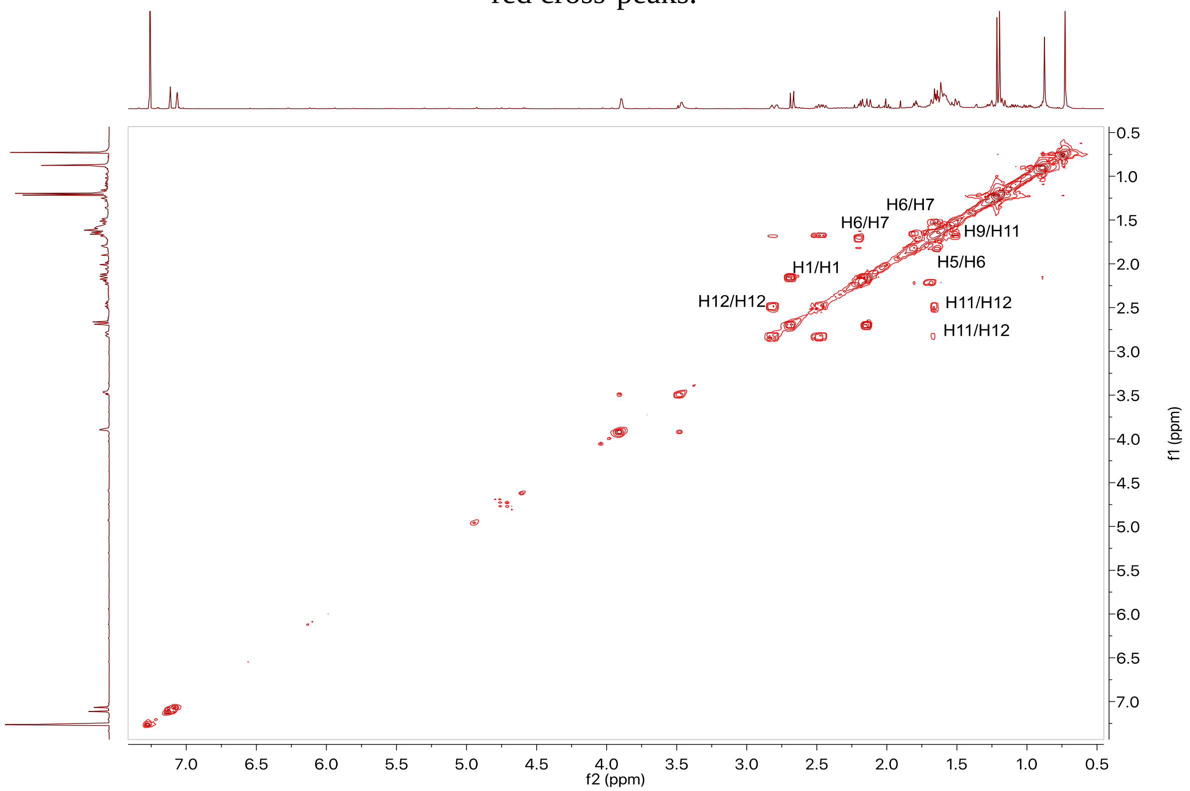

Figure S4. COSY spectrum of **1** (500 MHz, $CDCl_3$).

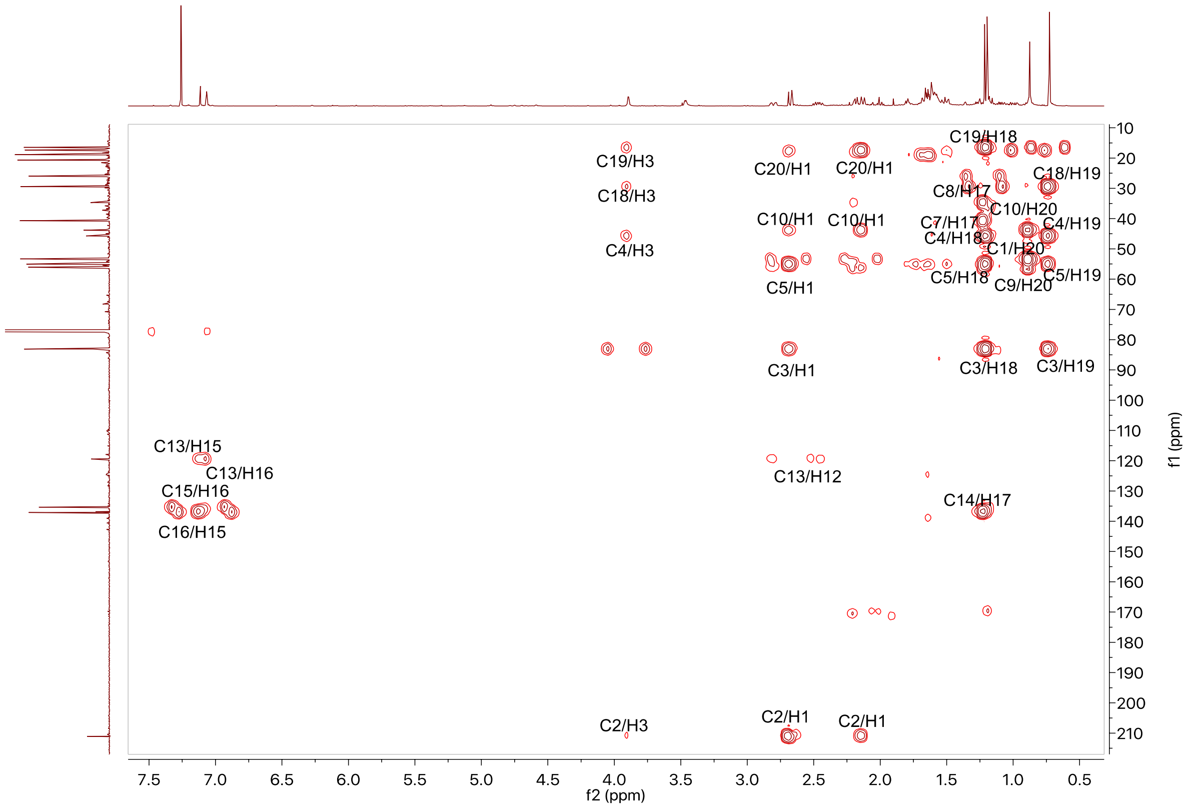

Figure S5. HMBC spectrum of **1** (500 MHz, $CDCl_3$).

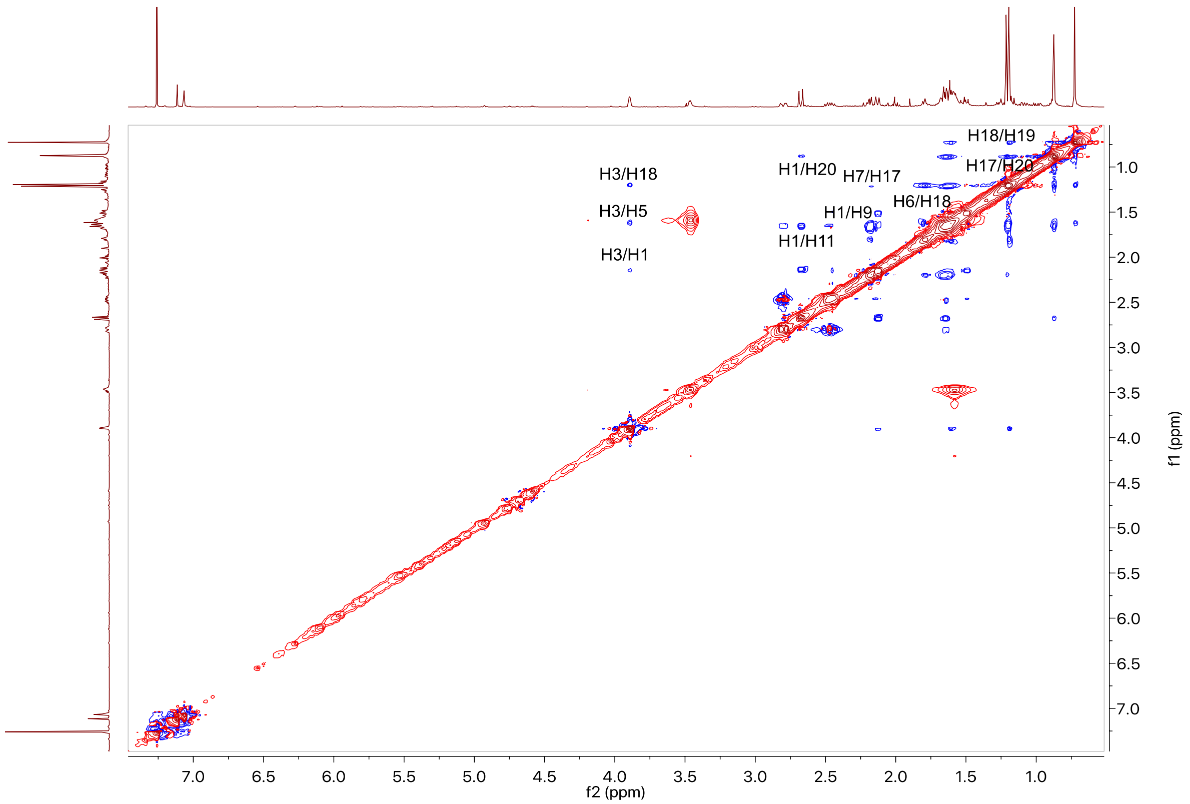

Figure S6. NOESY spectrum of **1** (500 MHz, $CDCl_3$).

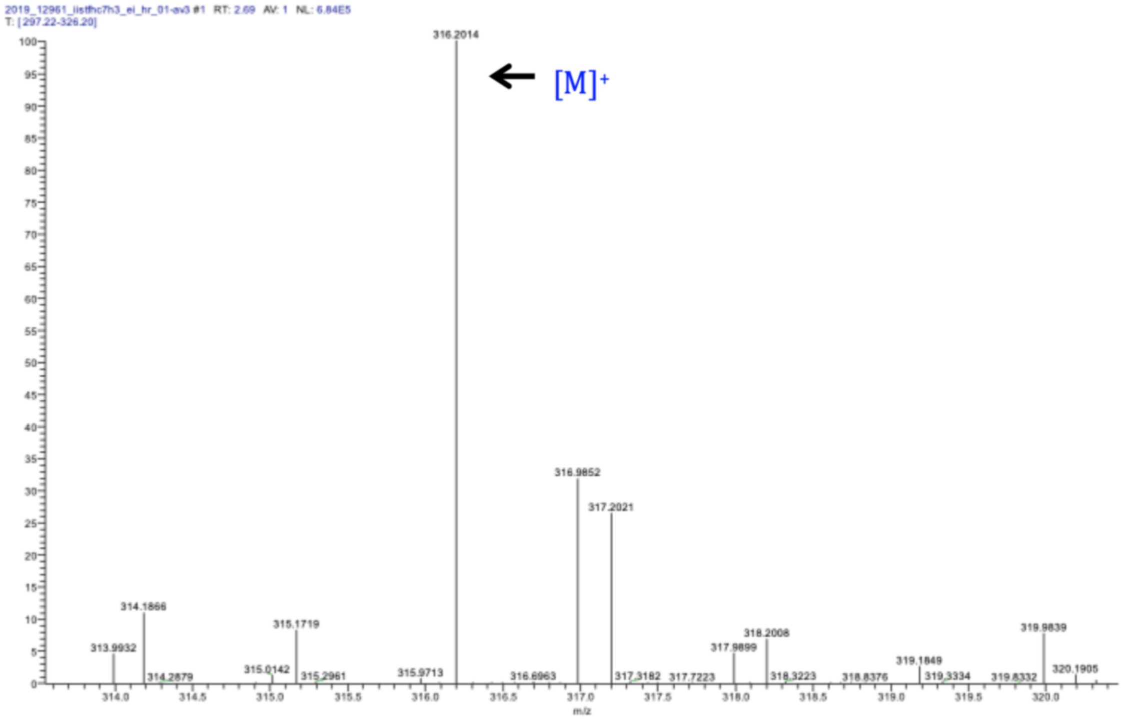

Figure S7. HREIMS of **1.**

Table S2. NMR data of **2** in $CDCl_3$ (125 MHz for ^{13}C and 500 MHz for ^{1}H).

no.	δ_C type	δ_H, mult. (*J* in Hz)	COSY	HMBC	NOESY
1	128.3, CH	6.54, s		2, 3, 5, 9	9, 11, 20
2	144.3, C				
3	201.2, C				
4	44.3, C				
5	54.5, C	1.80, m	6		
6	19.1, CH_2	1.67, m	5, 7		
7	40.4, CH_2	1.66, m-2.18, m	6		15
8	34.9, C				
9	51.7, CH	1.48, dd (11.8, 1.7)	11	5, 9	1
10	38.8, C				
11	18.8, CH_2	1.91, dt (7.0, 1.7)	9, 12	13	1
12	20.7, CH_2	2.51, dddd(16.2, 12.2, 7.0, 1.7)<br>2.83, ddt (16.2, 6.3, 1.5)	11	9, 13	16
13	119.5, C				
14	137.3, C				
15	135.0, CH	7.09, s		13, 16	7, 17
16	137.2, CH	7.06, s		13, 15	12
17	26.7, CH_3	1.28, s		7, 8, 9, 14	15
18	20.6, CH_3	1.16, s		3, 4, 5, 19	
19	27.3, CH_3	1.23, s		3, 4, 5, 18	
20	21.7, CH_3	1.22, s		1, 9, 10	1
OH		5.93, s		1, 2	

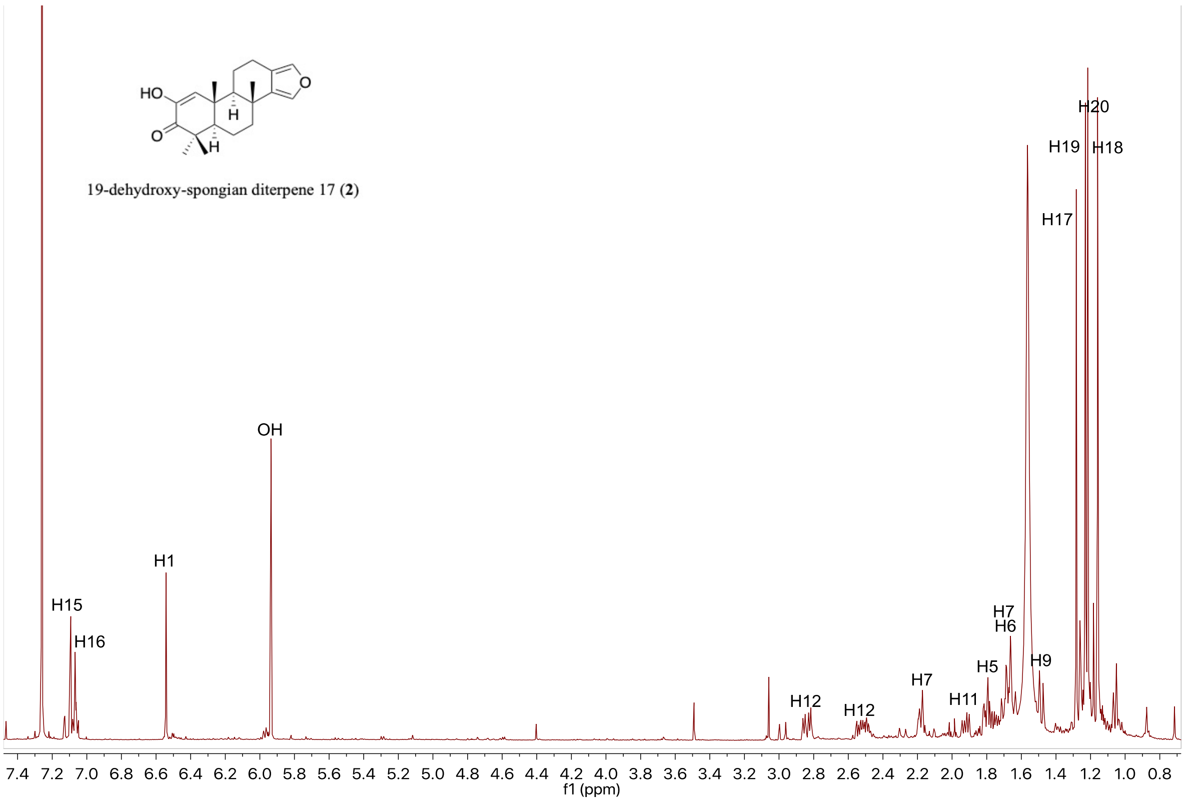

Figure S8. ^{1}H NMR spectrum of **2** (500 MHz, $CDCl_3$).

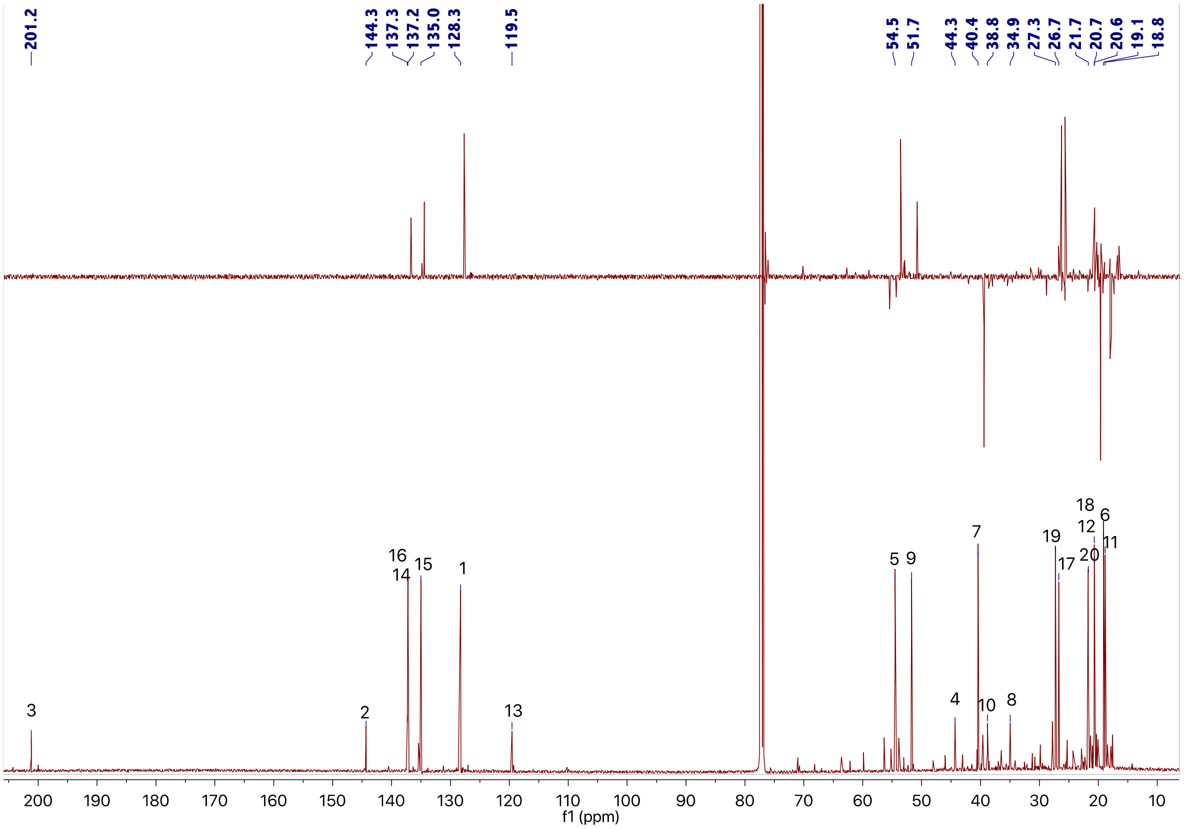

Figure S9. ^{13}C NMR and DEPT-135 spectrum of **2** (125 MHz, $CDCl_3$).

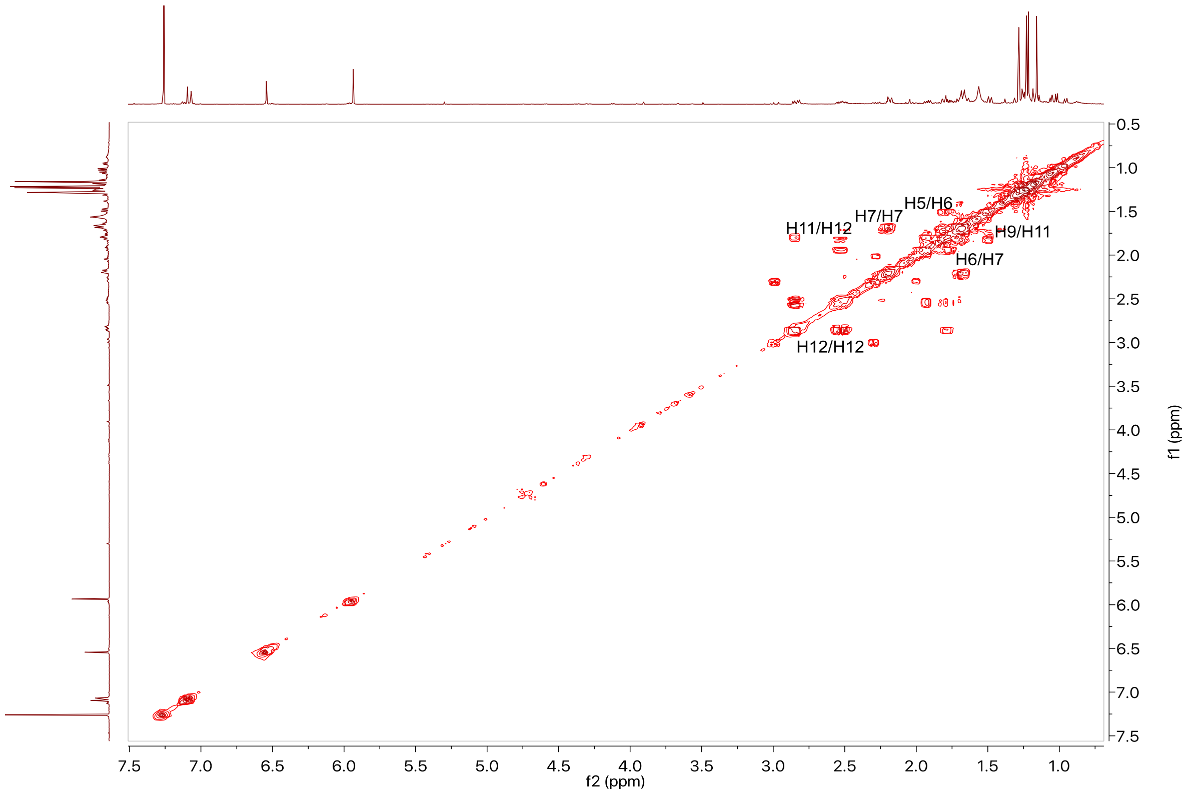

Figure S10. COSY spectrum of **2** (500 MHz, $CDCl_3$).

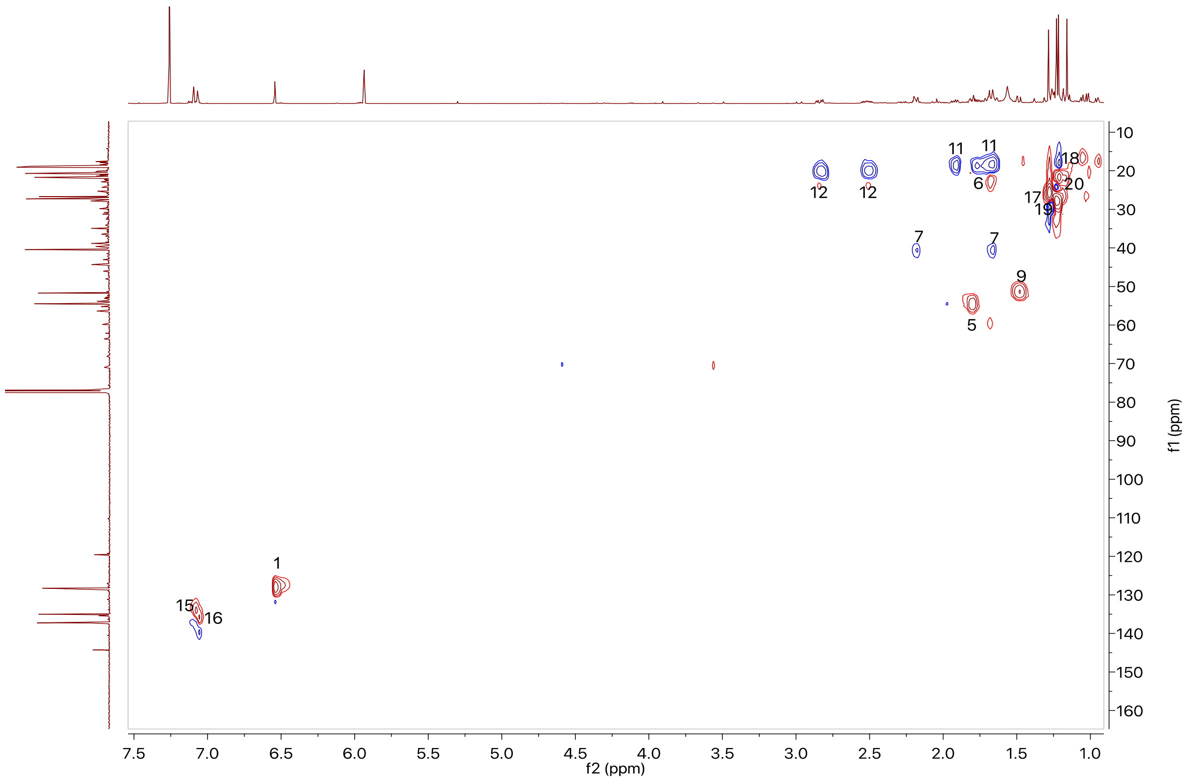

Figure S11. HSQC spectrum of **2** (500 MHz, $CDCl_3$). CH_2: blue cross-peaks and CH or CH_3: red cross-peaks.

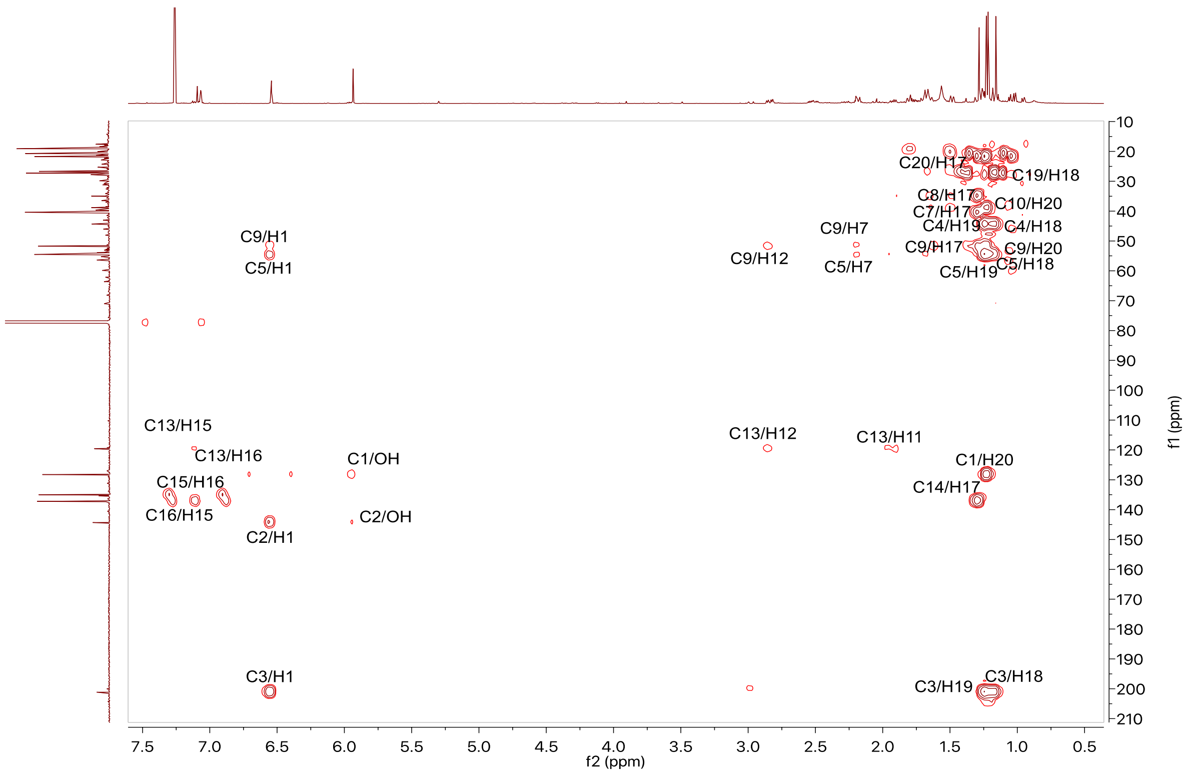

Figure S12. HMBC spectrum of **2** (500 MHz, $CDCl_3$).

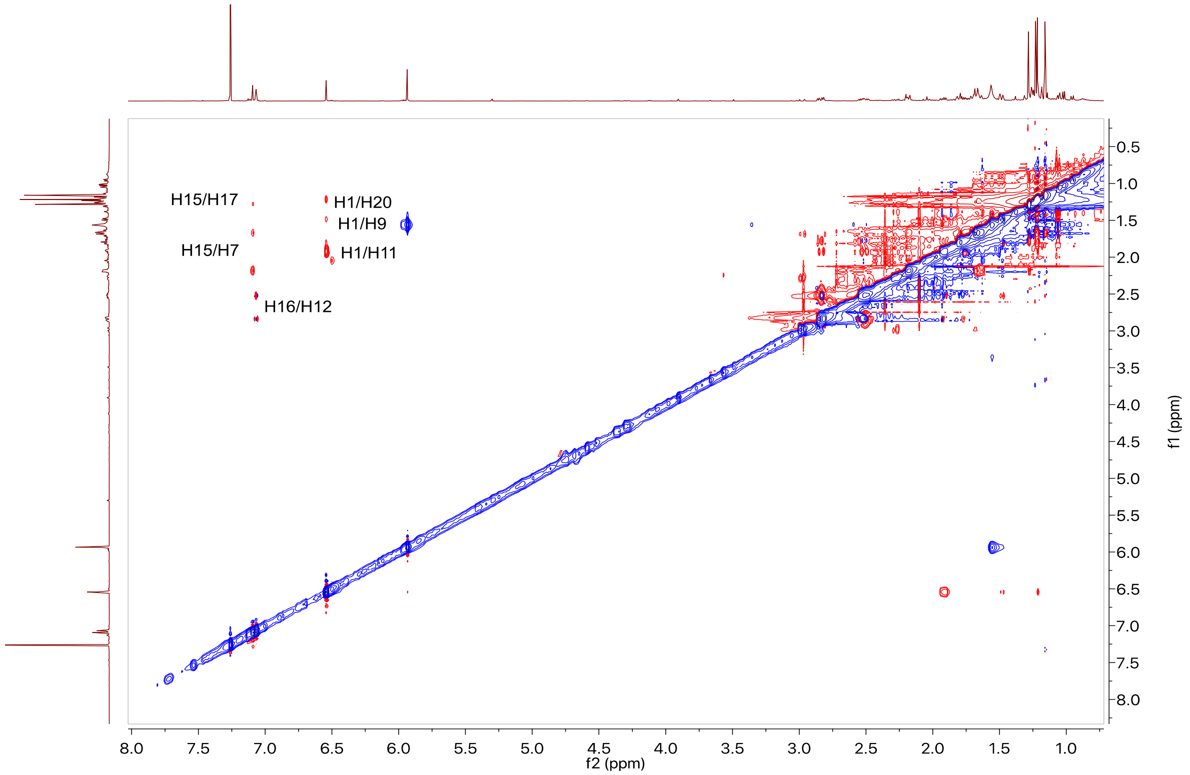

Figure S13. NOESY spectrum of **2** (500 MHz, $CDCl_3$).

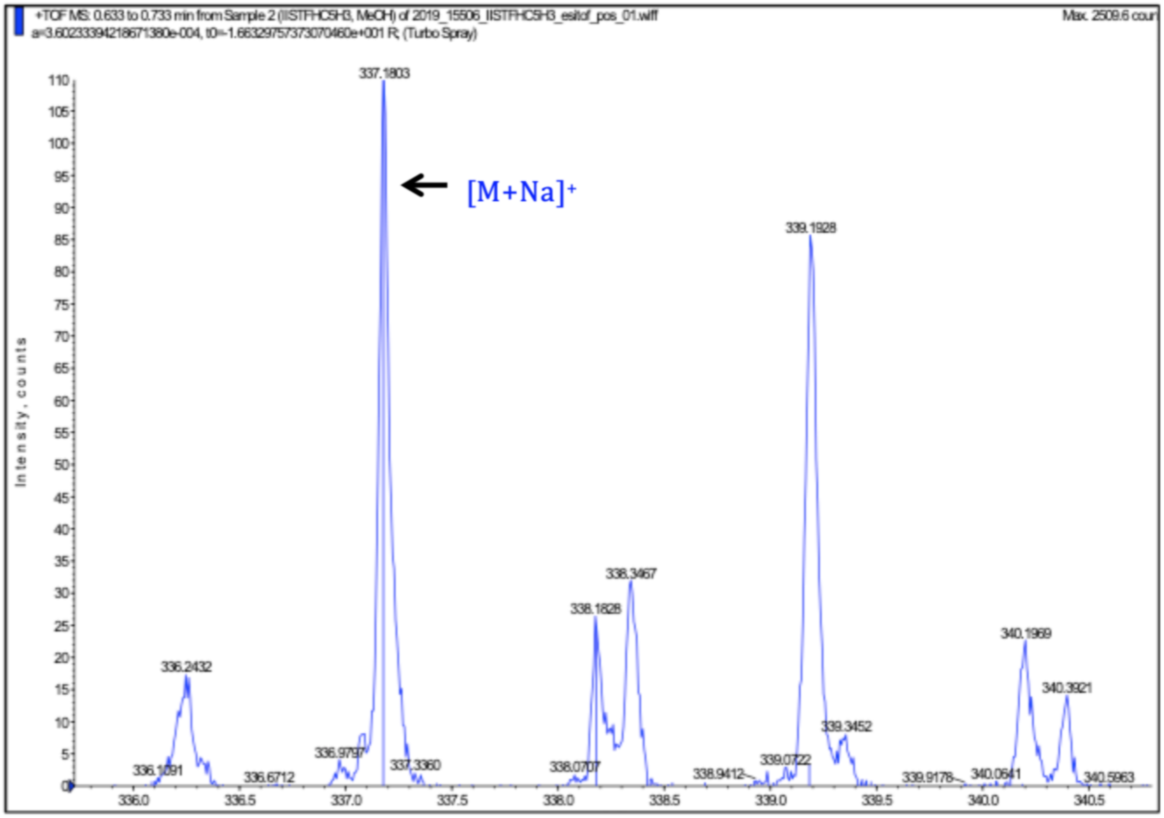

Figure S14. (+)-HRESIMS of **2**.

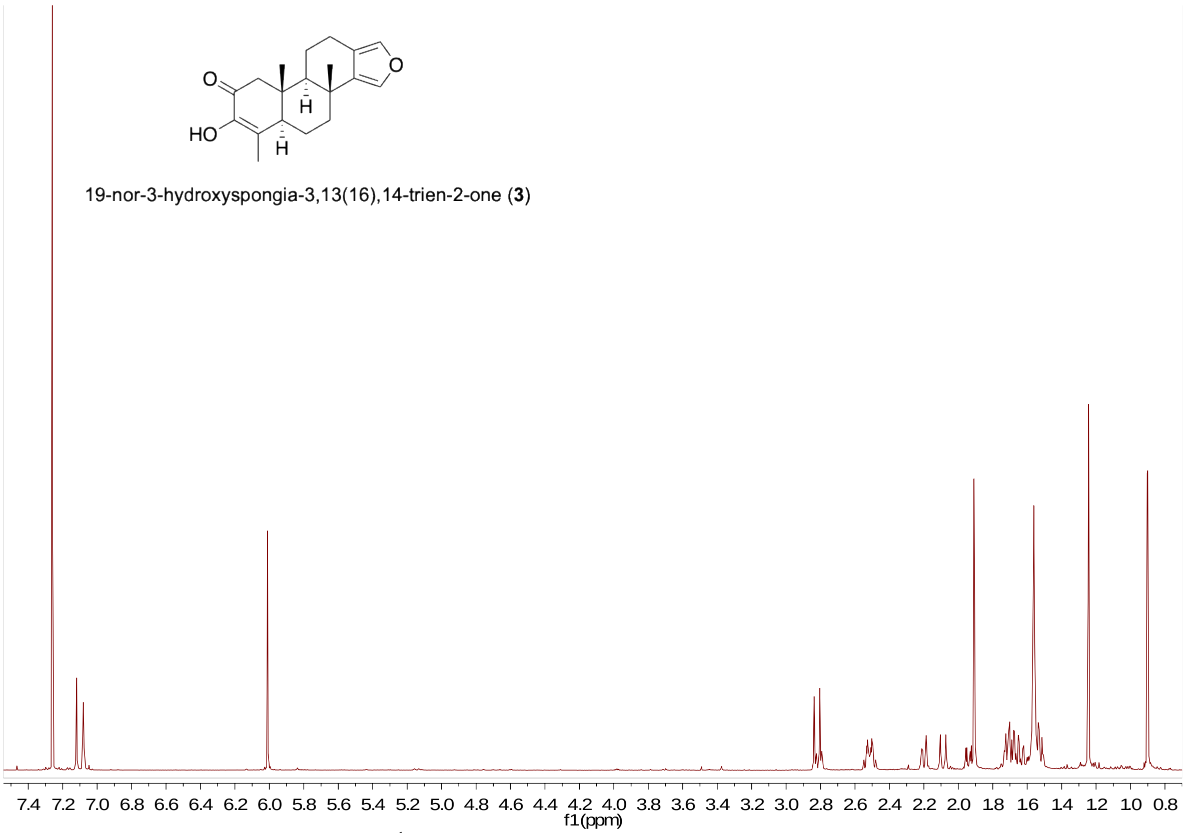

Figure S15. ^{1}H NMR spectrum of **3** (500 MHz, $CDCl_3$).

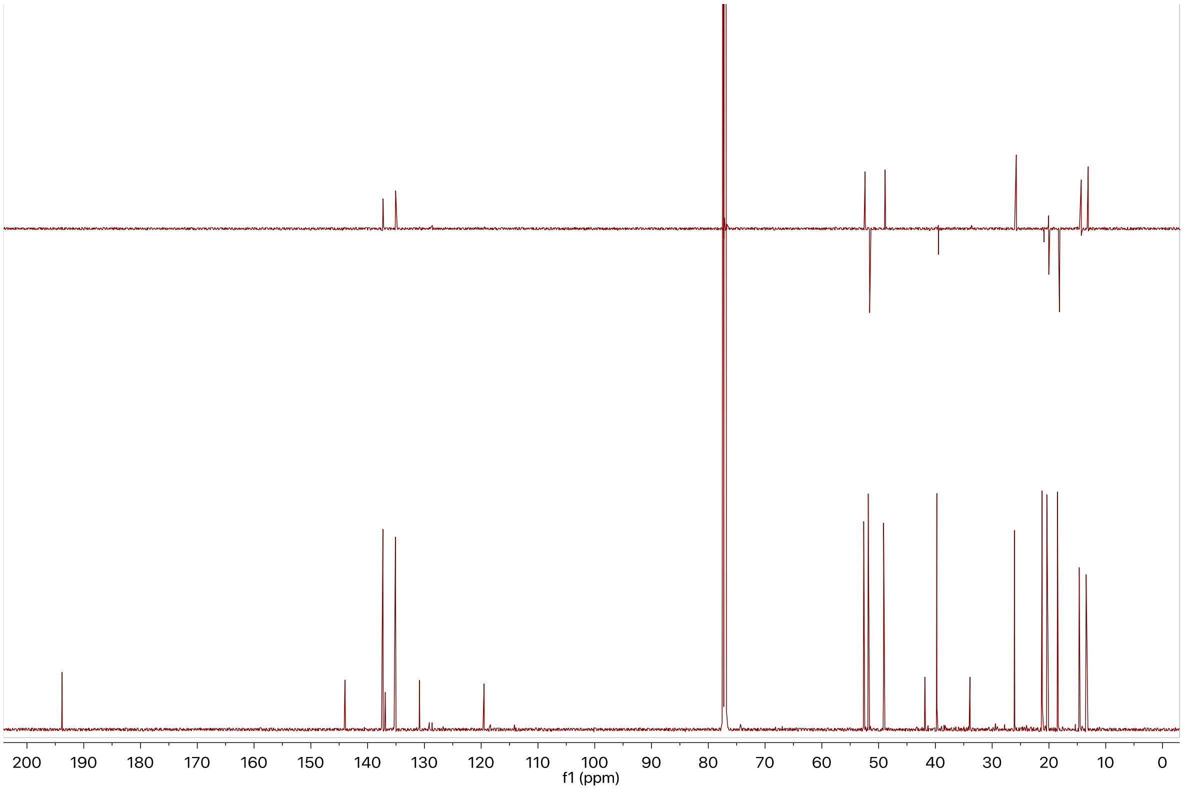

Figure S16. ^{13}C NMR and DEPT-135 spectra of **3** (125 MHz, $CDCl_3$)

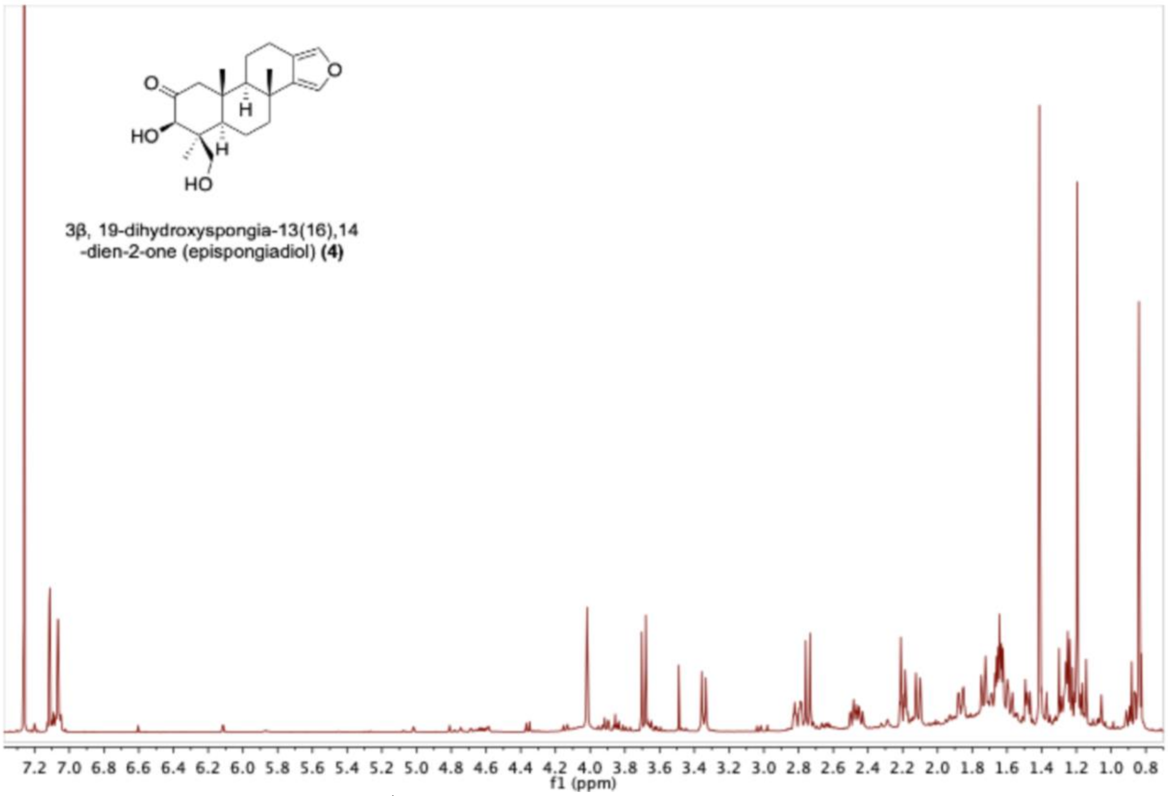

Figure S17. ^{1}H NMR spectrum of **4** (500 MHz, $CDCl_3$).

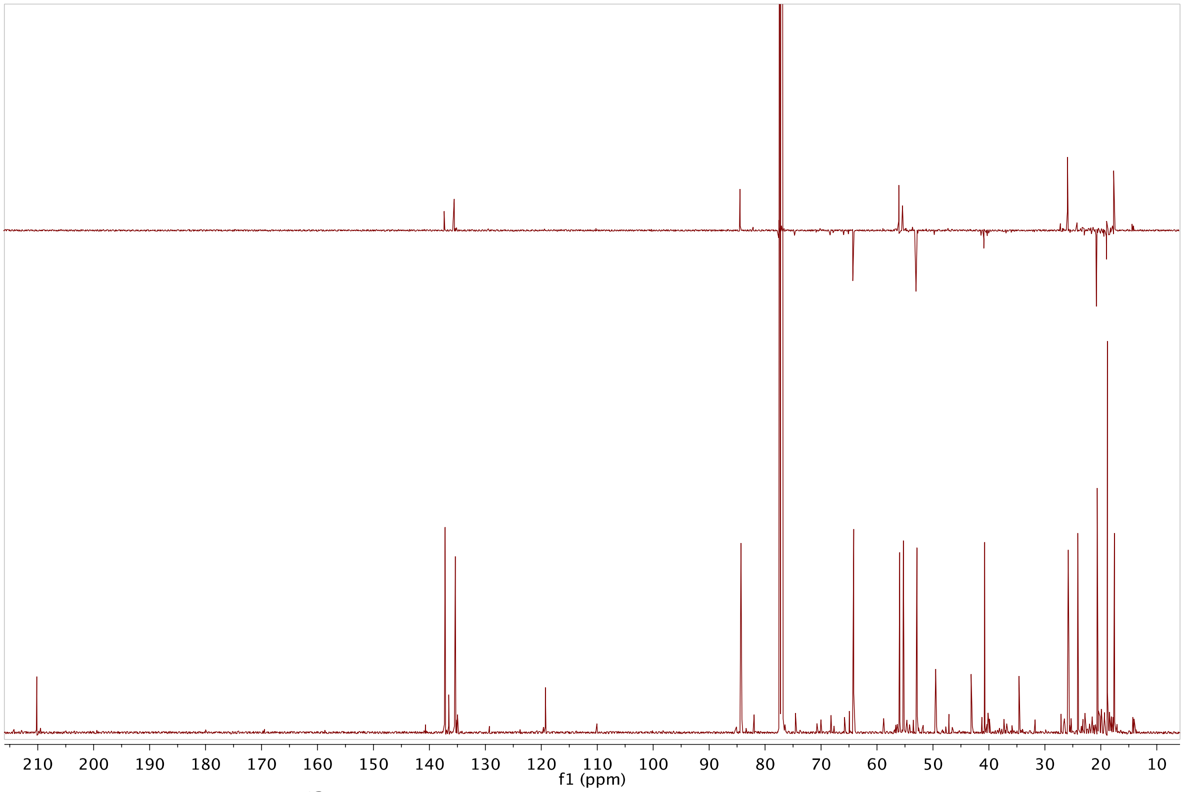

Figure S18. ^{13}C NMR and DEPT-135 spectra of **4** (125 MHz, $CDCl_3$).

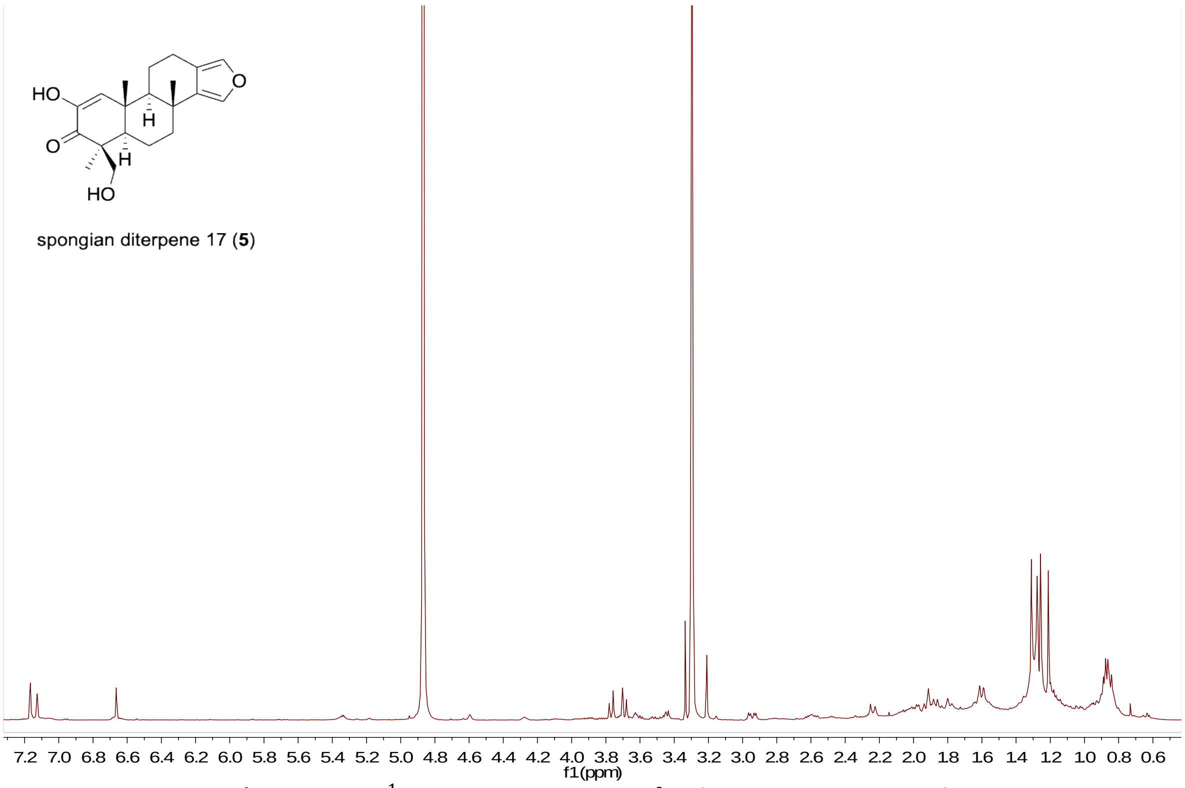

Figure S19. ^{1}H NMR spectrum of **5** (500 MHz, CH_3OH).

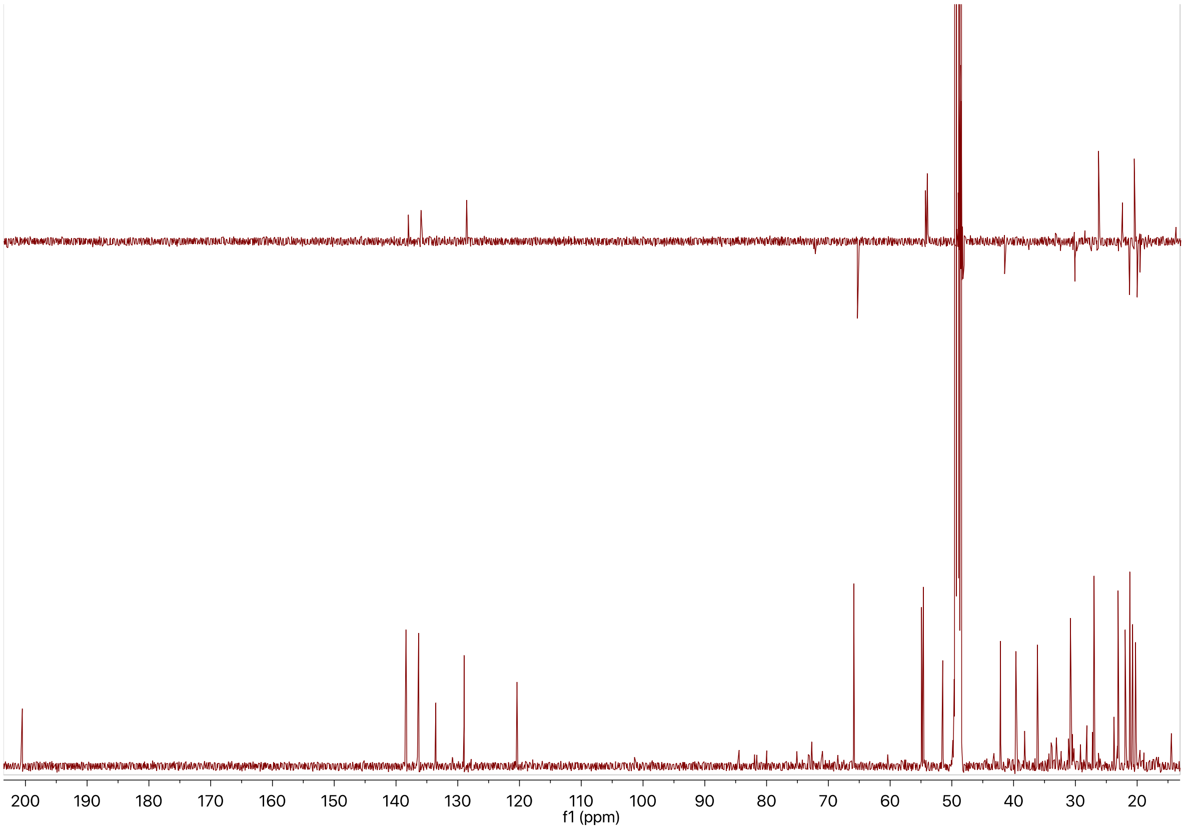

Figure S20. ^{13}C NMR and DEPT-135 spectra of **5** (125 MHz, CH_3OH).

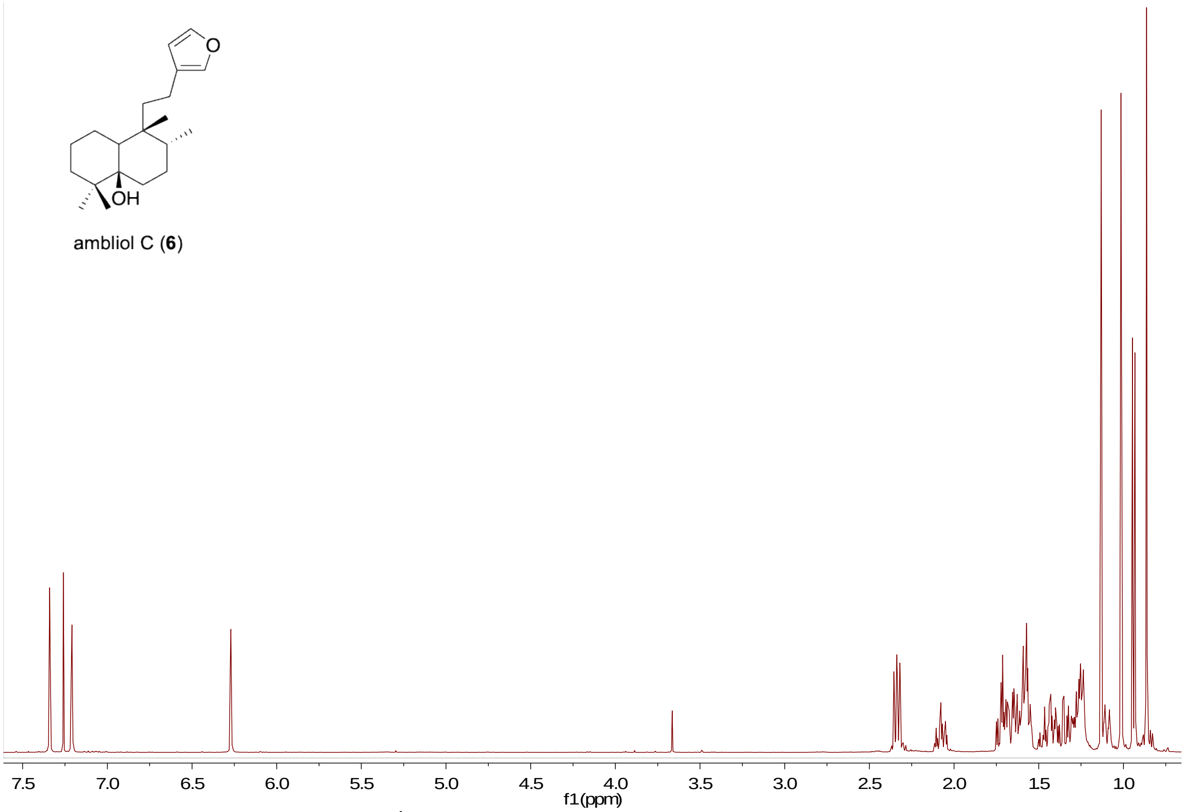

Figure S21. ^{1}H NMR spectrum of **6** (500 MHz, $CDCl_3$).

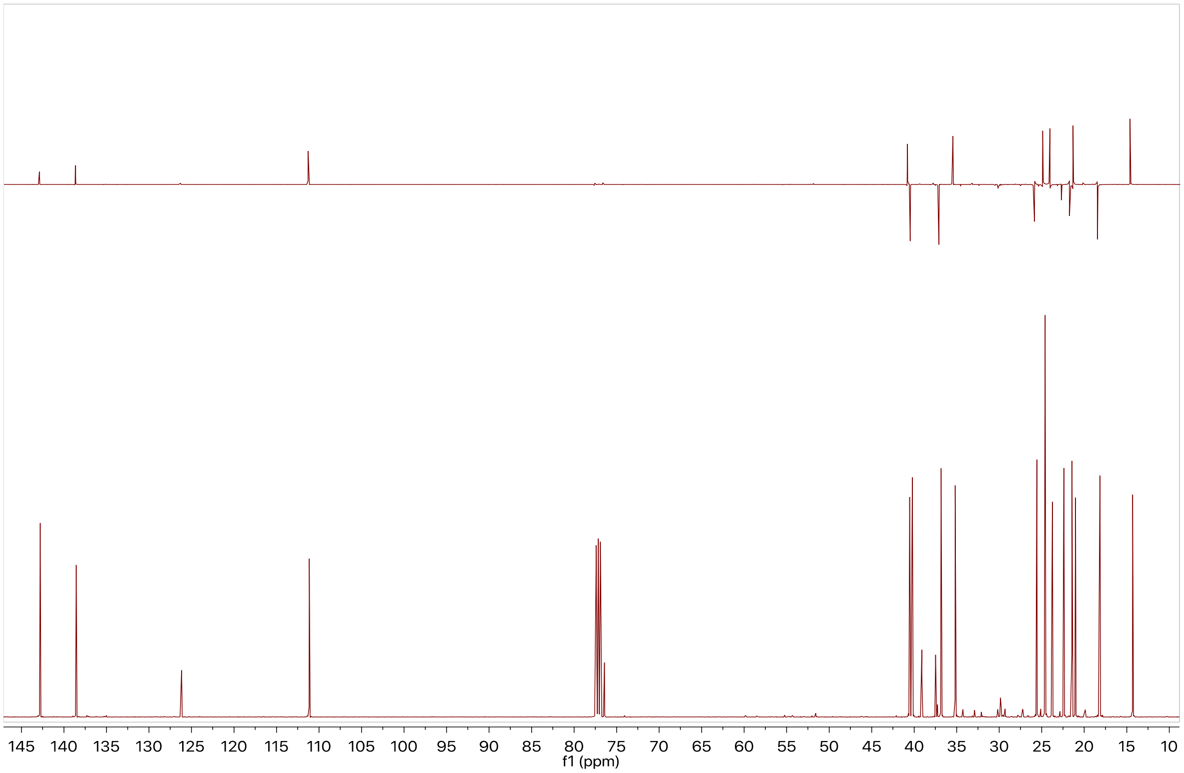

Figure S22. ^{13}C NMR and DEPT-135 spectra of **6** (125 MHz, $CDCl_3$).

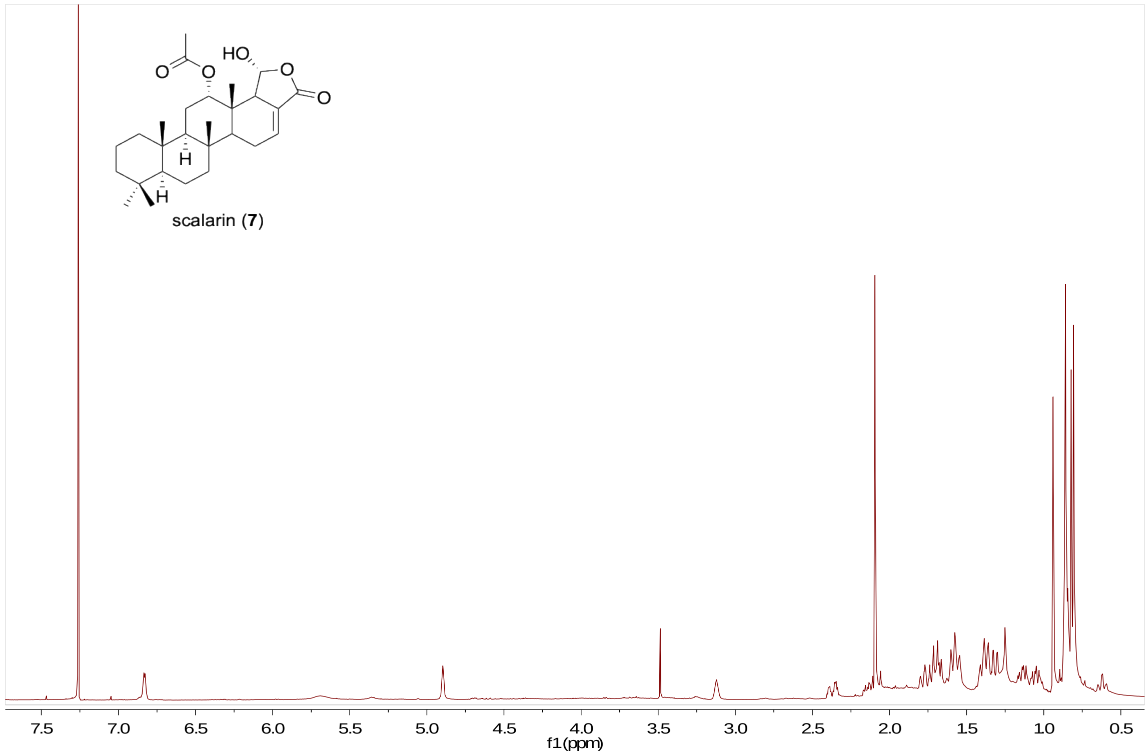

Figure S23. ^{1}H NMR spectrum of **7** (500 MHz, $CDCl_3$).

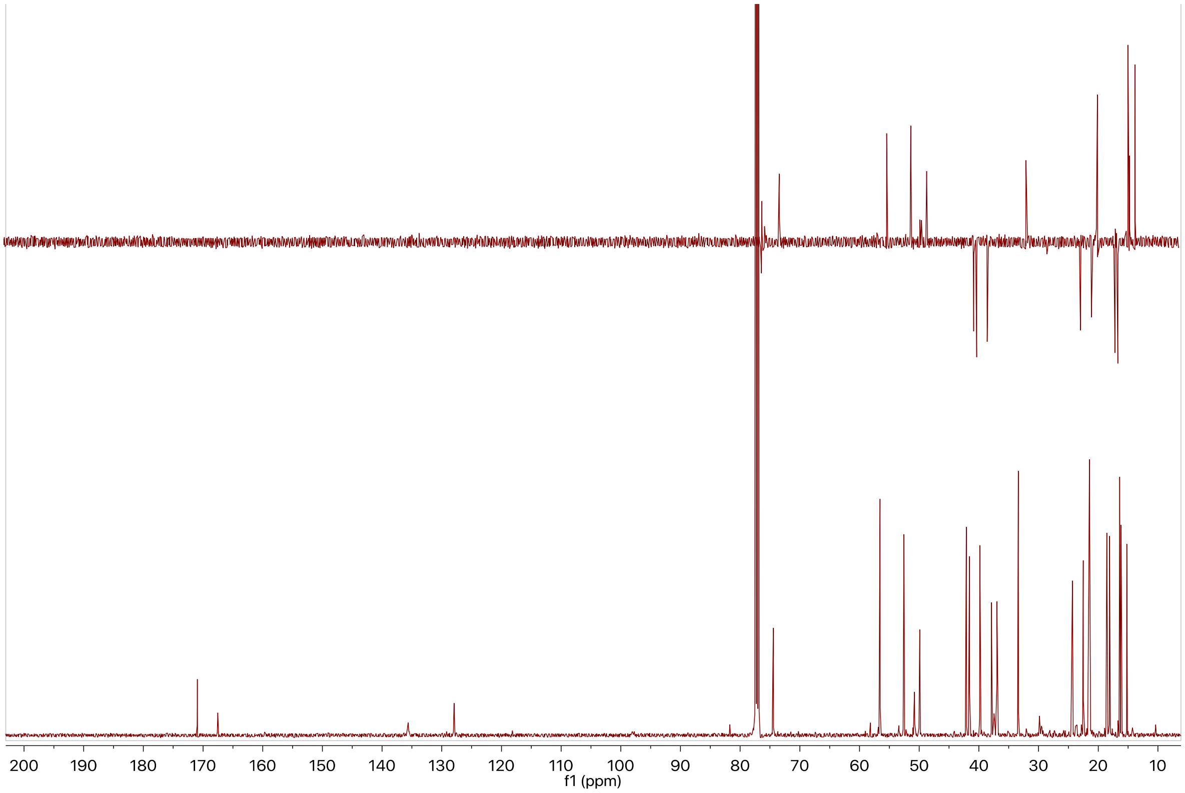

Figure S24. ^{13}C NMR and DEPT-135 spectra of **7** (125 MHz, $CDCl_3$).
